# Varicose projection astrocytes: Conserved reactive cells in brain pathology

**DOI:** 10.1126/sciadv.ady8204

**Published:** 2026-07-17

**Authors:** Caterina Ciani, Giulio Pistorio, Simona Giancola, Marika Mearelli, Francesca Emma Mongelli, Delaram Forouzeh, Luana Campos Soares, Marta Gonzalez Martin, Mandi E. Lichtenstein, Luca Mio, Elena Christou, Kyle Hastings, Anh Quynh Nguyen Do, Paula Ramos-Gonzalez, Carlos Matute, Ugne Kuliesiute, Sari Elena Dötterer, Gediminas Luksys, Saulius Rocka, Urte Neniskyte, Daniel C. Anthony, Marya Ayub, Cinzia Centelleghe, Maria Angeles Arevalo, Jean-Marie Graïc, Francesco Petrelli, Alexei Verkhratsky, Nunzio Iraci, Fabio Cavaliere, Carmen Muñoz-Ballester, Mootaz M. Salman, Francesco P. Ulloa Severino, Carmen Falcone

**Affiliations:** ^1^ Department of Neuroscience, Scuola Internazionale Superiore di Studi Avanzati (SISSA), Trieste, Italy.; ^2^ Center for Synaptic Neuroscience and Technology, Italian Institute of Technology, Genoa, Italy.; ^3^ Department of Physiology, Anatomy and Genetics, University of Oxford, Parks Road, Oxford OX1 3PT, UK.; ^4^ BHF Oxford Centre of Research Excellence, University of Oxford, Oxford, UK.; ^5^ Kavli Institute for NanoScience Discovery, University of Oxford, Oxford, UK.; ^6^ British Heart Foundation (BHF)–UK Dementia Research Institute (UK DRI) Centre for Vascular Dementia Research at the University of Oxford, Oxford, UK.; ^7^ Cajal Institute (CSIC), Madrid, Spain.; ^8^ Cajal Neuroscience Center, CSIC, Campus de Alcalá de Henares, Madrid, Spain.; ^9^ Department of Biological Sciences, University of Maryland Baltimore County, Baltimore, MD, USA.; ^10^ Department of Biological Sciences, Towson University, Towson, MD, USA.; ^11^ Achucarro Basque Center for Neuroscience, The Basque Biomodels Platforms for Human Research, Leioa, Spain.; ^12^ Department of Neuroscience, University of the Basque Country (UPV/EHU), Leioa, Spain.; ^13^ CIBERNED, Madrid, Spain.; ^14^ Institute of Biosciences, Life Sciences Center, Vilnius University, Vilnius, Lithuania.; ^15^ VU LSC-EMBL Partnership Institute for Genome Editing Technologies, Life Sciences Center, Vilnius University, Vilnius, Lithuania.; ^16^ Faculty of Medicine, Translational Health Research Institute, Vilnius University, Vilnius, Lithuania.; ^17^ Department of Pharmacology, University of Oxford, Oxford, UK.; ^18^ Department of Comparative Biomedicine and Food Science, University of Padua, Padua, Italy.; ^19^ CIBERFES, ISCIII, Madrid, Spain.; ^20^ Department of Biomedical Sciences, University of Lausanne, Lausanne, Switzerland.; ^21^ School of Pharmacy, University of Rome “Tor Vergata”, Rome, Italy.; ^22^ Faculty of Biology, Medicine and Health, The University of Manchester, Manchester, UK.; ^23^ Department of Biomedical and Biotechnological Sciences, University of Catania, Catania, Italy.

## Abstract

Astrocytes play essential roles in neuropathology. Human astrocytes exhibit unique properties, highlighting the importance of studying astrocytic responses in human models. Varicose projection astrocytes, previously considered exclusive to hominoids and a physiological type of astrocytes, were suggested to reflect pathological burden, albeit direct evidence linking them to neurological diseases has been lacking. Here, we demonstrate that varicose projection astrocytes also appear in other mammals and show, from four distinct human-based disease models, that varicose projection astrocytes are induced by neuroinflammation and characterized by distinctive subcellular features, indicating involvement in cellular stress responses, and their density is increased in aging and human neuropathology. Our findings establish varicose projection astrocytes as a reactive phenotype associated with neuropathology.

## Teaser

Varicose projection astrocytes emerge as a conserved reactive phenotype linked to inflammation and brain pathology.

## INTRODUCTION

Astrocytes are essential for maintaining brain homeostasis, including supporting synaptic transmission and modulating neuronal activity (*1*). Under pathological conditions, they undergo reactive astrogliosis, characterized by morphological and functional changes such as hypertrophy, process elongation, up-regulation of glial fibrillary acidic protein (GFAP) (*2*–*5*), or dynamic relocalization of proteins such as aquaporin-4 (*6*, *7*). This reactivity can be neuroprotective or detrimental, depending on the context (*8*–*10*). A significant trigger for astrocyte reactivity is neuroinflammation, involving pro-inflammatory molecules like cytokines and chemokines (*11*–*14*). Microglia play a pivotal role by releasing factors such as tumor necrosis factor–α (TNF-α), interleukin-1β (IL-1β), and complement component 1q (C1q), which trigger astrogliosis (*9*, *15*, *16*). When astrocyte reactivity persists or is dysregulated, it may contribute to chronic neuropathological states (*3*, *11*, *12*, *14*). The consequences of astrocyte reactivity are widespread and heterogeneous, ranging from synaptic damage and reduced neuronal connectivity to increased immune cell infiltration, which amplifies inflammation and disrupts classical astrocyte functions. These malfunctions were implicated under multiple neurological and psychiatric conditions (*1*, *9*, *14*, *16*). Despite extensive studies in rodent models, the specific contributions of astrocytes to human pathology remain inadequately understood, partly due to species-specific differences (*17*–*20*). Varicose projection astrocytes represent a distinct astrocyte population that were originally contemplated to be unique to humans and other hominoids (*21*, *22*). Varicose projection astrocytes are defined by their characteristic morphology: Somata localized in deep cortical layers (V, VI, and white matter) sends long, GFAP-positive processes containing evenly spaced varicosities (bead-like dilatations) that span in multiple directions without territorial segregation (tiling) characteristic of protoplasmic astrocytes (*17*, *21*). Although pronounced process elongation is a prominent feature of varicose projection astrocytes in many in vivo descriptions, subsequent observations in human tissue have reported varicosities occurring on astrocytic processes that are not always markedly longer than other processes emerging from the same cell. Thus, although elongation is a characteristic feature in situ, it does not appear to be an absolute requirement for the presence of varicosities. On the basis of early description in postmortem tissue and surgical resections, varicose projection astrocytes were considered a physiological subtype of human/hominoid astrocytes with unclear functional significance (*21*, *22*).

However, this interpretation is problematic. Varicose projection astrocytes have been previously described in aged or diseased human brains and not always found in all the samples within the same species (*21*, *22*). This raised the possibility that varicose projection astrocytes may not represent a stable physiological cell type but rather an astrocytic state emerging under or as a consequence of pathological conditions.

We therefore hypothesized that varicose projection astrocytes might arise in response to neuropathology (and more specifically to neuroinflammation) and could appear across different mammalian species, not only humans.

Here, we demonstrate that varicose projection astrocytes are not hominoid specific but can also be found in other mammalian species, such as mice and tigers. In addition, we demonstrate that varicose projection astrocytes are induced by pro-inflammatory cytokines in human-based models, including human-induced pluripotent stem cell (hiPSC)–derived and human neural stem cell (hNSC)–derived astrocytes and cerebral organoids, as well as in mouse astrocyte cultures, and that this induction is reversible upon cytokine withdrawal. Last, we establish the pathological relevance of varicose projection astrocytes by documenting their increased density in human neurodegenerative diseases such as Alzheimer’s disease (AD), Parkinson’s disease (PD), multiple sclerosis (MS), and epilepsy, as well as demonstrating their accumulation with aging. Furthermore, we found that varicose projection astrocyte density is increased in mouse animal models of traumatic brain injury and of lipopolysaccharide (LPS)–induced neuroinflammation.

Our findings indicate that varicose projection astrocytes represent an evolutionary conserved reactive phenotype emerging in response to pathological conditions, rather than a physiological subtype. This enhances our understanding of astrocyte reactivity in brain pathology and positions varicose projection astrocytes as potential biomarkers or therapeutic targets in neuroinflammatory and neurodegenerative disorders.

## RESULTS

### Varicose projection astrocytes are not hominoid specific but are present across mammals

**Fig. 1.**
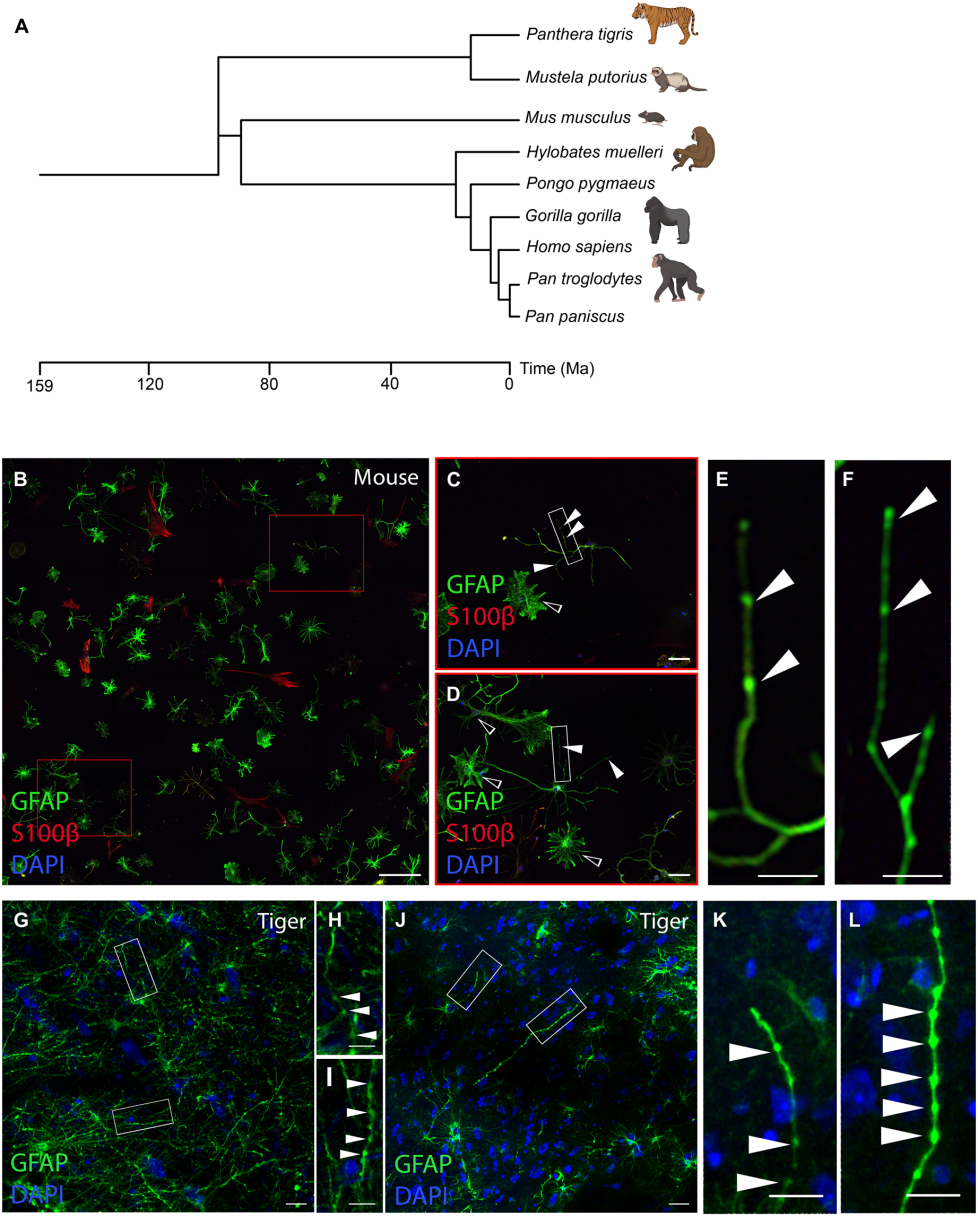
Varicose projection astrocytes are not hominoid specific but are found across mammals. (**A**) Evolutionary tree showing the mammalian species that have been observed to show varicose projection astrocytes and illustrating their phylogenetical relationships. Ma, million years ago. Cladogram created with the help of the tool at: https://phylot.biobyte.de/. Icons of different animals were created with BioRender [Created in BioRender. Falcone, C. (2026) https://BioRender.com/saer3rj]. (**B** to **F**) Representative IF images of ESC-derived mouse astrocytes stained for GFAP (green), S100β (red), and DAPI (blue) (*n* = 3). (C and D) Higher magnification of the boxed regions in (B), highlighting varicosities (white filled arrowheads). Empty arrowheads point to non–varicose projection astrocytes. (E and F) Zoomed-in images of the boxed regions in (C) and (D), respectively. (**G** to **L**) GFAP immunostaining of postmortem prefrontal cortex tissue from a tiger (*P. tigris*), showing reactive astrocytes and varicose projection astrocytes (*n* = 5). (H, I, K, and L) Higher magnification of the boxed regions in (G) and (J), with white filled arrowheads pointing to varicosities. Scale bars, 100 μm (B), 25 μm [(C) and (D)], 50 μm [(G) and (H)], and 25 μm [(E), (F), (H), (I), (K), and (L)].

Varicose projection astrocytes were thought to be exclusive to humans and other hominoids as they were identified only in primate brains. However, our observations reveal their presence in other mammalian species, including mouse (Fig. 1, A to F), indicating that varicose projection astrocytes are not restricted to primates. We also identified varicose projection astrocytes in tiger brains (*Panthera tigris*; Fig. 1, G to L), further supporting their presence in gyrencephalic carnivores, as previously reported for ferret (*23*). This suggests that varicose projection astrocytes may represent a conserved astrocytic type across diverse mammalian species, rather than being exclusive to primates. Notably, similarly to previous observations in hominoids, varicose projection astrocytes in other mammals are scattered and individual specific (for example, varicose projection astrocytes were present in three of five samples of tigers), meaning that not all individuals from the same species have them. This pattern further supports our hypothesis that varicose projection astrocytes are not a distinct physiological subtype but rather emerge under specific brain conditions, possibly reflecting accumulated pathological burden. Given this, we tested whether neuropathology, and neuroinflammation in particular, may drive varicose projection astrocyte formation.

### Varicose projection astrocytes are induced by pro-inflammatory cytokines in human astrocytes in vitro

To test our hypothesis, we differentiated human astrocytes from hiPSCs in vitro, following an adapted protocol (see Materials and Methods). The astrocytic identity of cultured cells was confirmed by immunostaining for canonical markers (S100β, SOX9, AQP4, and Kir4.1), with >92% of cells positive for SOX9, AQP4, and Kir4.1 (fig. S1). We treated these astrocytes with the pro-inflammatory cytokines IL-1β and TNF-α, either individually or in combination, at a concentration of 100 ng/ml for each cytokine (see Materials and Methods). We evaluated morphological and reactive changes in astrocytes upon these different treatments compared to the untreated control at different time points (1, 24, and 72 hours and 5 and 7 days). At the 1-hour time point, we assessed astrocyte reactivity by immunofluorescence (IF) staining for nuclear factor κB p65 (NF-κB p65), a transcription factor activated during acute inflammation. In untreated astrocytes, the NF-κB complex remained cytoplasmic (fig. S2, A to E). Following cytokine treatment, NF-κB p65 translocated to the nucleus, as shown by IF (fig. S2, A to E). We quantified the extent of nuclear translocation by calculating the nuclear-to-cytoplasmic intensity ratio and found the strongest NF-κB translocation under combined IL-1β and TNF-α treatment, with a threefold increase compared to controls (fig. S2A, IL-1β versus Ctr: + 69.6%, *P* = 0.0339; TNF-α versus Ctr: + 240.9%, *P* < 0.0001; IL-1β/TNF-α versus Ctr: + 302.2%, *P* < 0.0001).

We further confirmed astrocyte reactivity by examining astrocyte morphology at later time points using GFAP immunostaining. By day 7 (D7), astrocytes exhibited significant morphological changes, including soma enlargement and increased number of processes. These changes were evident in IL-1β–treated cells (fig. S2E), TNF-α–treated cells (fig. S2E), and most prominently in the cells treated with both cytokines (fig. S2E), with the volume of GFAP^+^ astrocytes significantly higher compared to control (volume of GFAP^+^ astrocytes after 7 days of treatment: Ctr = 13,220.33 ± 1323.90 μm^3^, IL-1β + TNF-α = 17,433.89 ± 630.46 μm^3^, *P* < 0.04; fig. S2D).

Next, we investigated the presence of varicose projection astrocytes in these cultures through GFAP and S100β immunostaining at the same time points. Given the differences between astrocyte morphology in vitro and in intact tissue, varicose projection astrocytes in culture were identified on the basis of the presence of regularly spaced, bead-like varicosities along discernible astrocytic processes, irrespective of absolute process length. In this context, process elongation was not used as an exclusionary criterion, as dissociated astrocytes rarely develop the extreme process lengths observed in vivo. We documented the appearance of varicose projection astrocytes after 5 and 7 days of cytokine treatments under the IL-1β (fig. S3, A, B, E, F, and I to Jj), TNF-α (fig. S3, A, B, E, F, and M to Nn), and combined treatment conditions (fig. S3, A, B, E, F, and Q to Rr). In contrast, varicose projection astrocytes were rare or absent in the control cultures at D7 and were not observed under any condition before D5 (fig. S4, A, B, C, D, G, H, K, L, O, and P). Quantification revealed up to a 45-fold increase in varicose projection astrocytes under the combined treatment condition compared to controls, suggesting that the synergy between IL-1β and TNF-α was most effective in inducing varicosities [% varicose projection astrocytes/total astrocytes in culture: Ctr = 0.07 ± 0.07%, IL-1β = 1.51 ± 0.60%, TNF-α = 1.07 ± 0.12%, IL-1β + TNF-α = 3.20 ± 0.32%, with *P*(Ctr versus I versus IL-1β) < 0.04, *P*(Ctr versus TNF-α) < 0.001, *P*(Ctr versus IL-1β + TNF-α) < 0.0003; fig. S3B]. Despite the increase, the overall frequency of varicose projection astrocytes remained low, consistent with previous findings (*21*) (fig. S2B).

**Fig. 2.**
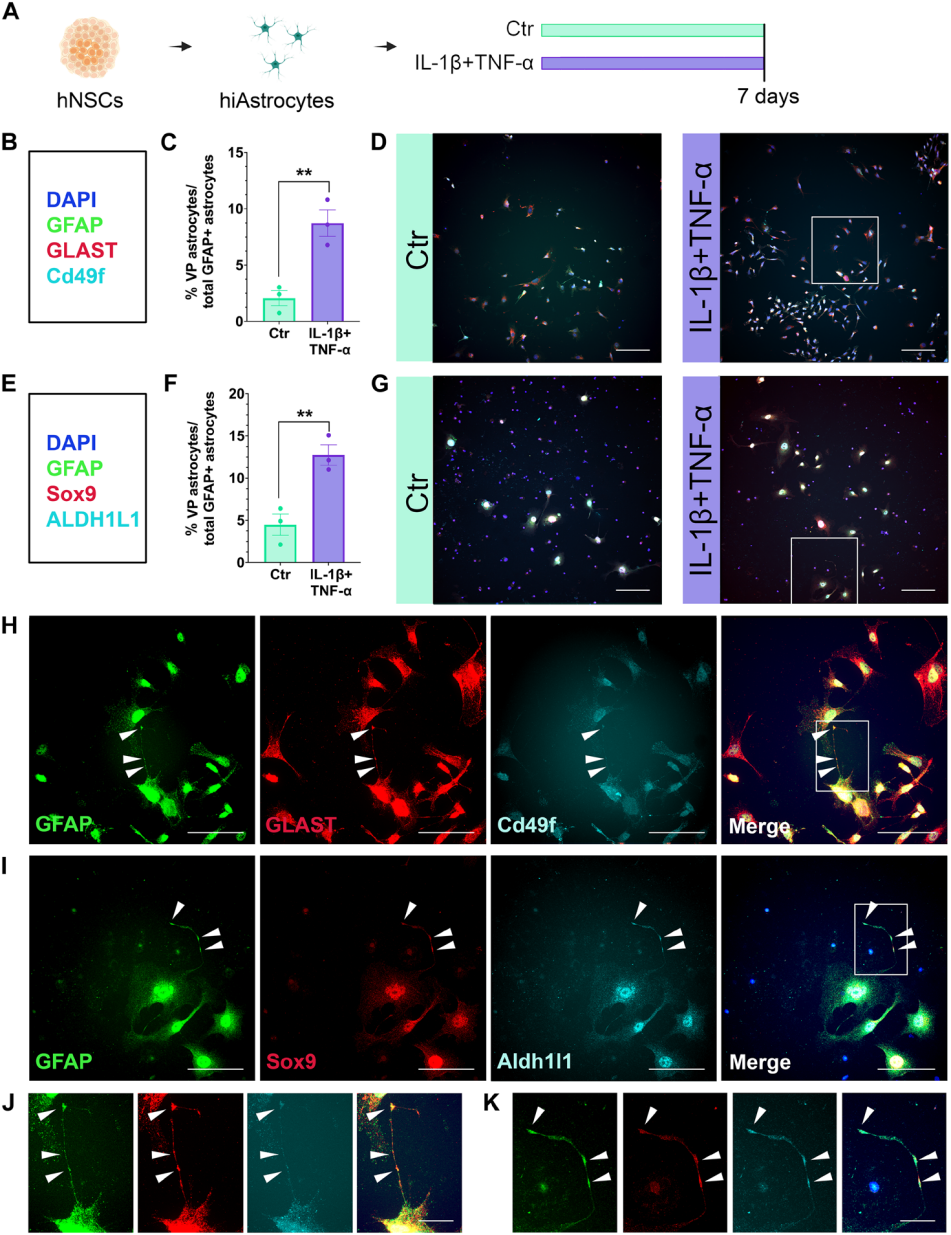
Varicose projection astrocytes are induced by pro-inflammatory cytokines in human astrocytes. (**A**) Schematic representation of the experimental protocol, created with BioRender [Created in BioRender. Falcone, C. (2026) https://BioRender.com/brgrkfp]. hNSC-derived astrocytes were treated with IL-1β + TNF-α for 7 days, followed by IF analysis in two parallel experiments. hiAstrocytes, human-induced astrocytes. (**B**) Marker combination used in IF for experiment 1. (**C**) Quantification of GFAP^+^/GLAST^+^/CD49f^+^ varicose projection astrocytes as a percentage of total GFAP^+^ astrocytes. Data are means ± SEM; statistical significance was assessed by the unpaired two-tailed *t* test (**P < 0.01). (**D**) Representative IF images of astrocytes under control (Ctr) and IL-1β + TNF-α–treated conditions (at a 20× objective). (**E**) Marker combination used in IF for experiment 2. (**F**) Quantification of GFAP^+^/Sox9^+^/Aldh1l1^+^ varicose projection astrocytes over total GFAP^+^ astrocytes. Data are means ± SEM; statistical significance was assessed by the unpaired two-tailed *t* test (**P < 0.01). (**G**) Representative IF images of astrocytes under control and IL-1β + TNF-α–treated conditions (20× objective). (**H**) Higher magnification of the boxed region in (D), IL-1β + TNF-α condition. Green: GFAP, GLAST (red), CD49f (cyan). (**I**) Higher magnification of the boxed region in (G), IL-1β + TNF-α condition. Green: GFAP; red: Sox9; cyan: Aldh1l1. (**J** and **K**) Further magnification of varicose processes from (H) and (I), respectively. Scale bars, 50 μm [(D), (G), (H), and (I)] and 10 μm [(J) and (K)]. White arrowheads indicate varicosities.

To further strengthen the identification of these cells as bona fide astrocytes, we repeated the cytokine treatment experiment in an independent astrocyte model derived from hNSCs. In this system, cultures were treated with the combined IL-1β + TNF-α condition (100 ng/ml each) for 7 days, matching the paradigm that most robustly induced varicosities in the previous experiments. We performed two independent experiments in parallel incorporating additional astrocytic markers to assess lineage specificity, including EAAT1/GLAST and CD49f (*24*) in one experiment, as well as SOX9 and Aldh1l1 in the other one. As in hiPSC-derived astrocytes, we observed a significant increase in varicose projection astrocytes following cytokine treatment. Quantification revealed an increase in GFAP^+^/GLAST^+^/CD49f^+^ varicose projection astrocytes compared to controls (Ctr = 2.06 ± 0.68%, IL-1β + TNF-α = 8.73 ± 1.17%; Fig. 2, A, B to D, H, and J), as well as in GFAP^+^/SOX9^+^/Aldh1l1^+^ varicose projection astrocytes (Ctr = 4.49 ± 1.25%, IL-1β + TNF-α = 12.74 ± 1.21%; Fig. 2, A, E to G, I, and K). Notably, all identified varicose projection astrocytes were consistently positive for these additional astrocytic markers, supporting their astrocyte identity and excluding contamination by other cell types.

To further validate the link between pro-inflammatory stimulation and varicose projection astrocytes, we repeated these experiments in mixed cultures containing mature astrocytes and neurons, thereby providing a more physiologically relevant environment. First, we confirmed astrocyte identity by immunostaining the cultures for the astrocyte markers GFAP, ALDH1L1, AQP4, Kir4.1, EAAT1/GLAST, GS, SOX9, and vimentin (figs. S4 and S5) and for neuronal marker MAP2 (fig. S6A). We optimized cytokine concentrations for mixed culture to maintain cell viability, with each cytokine (10 ng/ml) identified as the optimal dose for inducing astrocyte reactivity (concentrations tested for each cytokine were 1, 10, and 30 ng/ml for 7 days; fig. S6). To assess the appearance of varicose projection astrocytes in mixed cultures upon treatments with IL-1β (10 ng/ml), TNF-α (10 ng/ml), or both combined, we immunostained the cells for GFAP and S100β, as before. We found a higher number of varicose projection astrocytes in the cytokine-treated samples compared to control samples, where the presence of varicosities was much lower, with a more pronounced increase under the combined treatment condition (fig. S7). These findings together provide the proof of principle that varicose projection astrocytes are not a physiological subtype of astrocytes, but their presence might be due to neuroinflammatory conditions.

### Varicose projection astrocytes reverse after removing the cytokine exposure

To determine whether the presence of varicose projection astrocytes in human astrocyte cultures is reversible, we conducted a cytokine withdrawal experiment. As in the experiment described in fig. S3, we treated hiPSC-derived astrocytes with a combination of pro-inflammatory cytokines IL-1β and TNF-α, at 100 ng/ml each (fig. S8A). After 1 week, we confirmed the presence of varicose projection astrocytes (fig. S8, B and C, second panel). We then removed the cytokines from the medium and cultured the astrocytes for an additional week. Notably, the number of varicose projection astrocytes significantly decreased under the cytokine withdrawal condition {% varicose projection astrocytes/total astrocytes in culture: Ctr(7 days) = 0.15 ± 0.09%, IL-1β + TNF-α(7 days) = 2.34 ± 0.49%, Ctr(14 days) = 0.04 ± 0.02%, IL-1β + TNF-α(withdrawal at 14 days) = 0.29 ± 0.02%, with *P*[IL-1β + TNF-α(7 days) versus IL-1β + TNF-α(withdrawal at 14 days)] < 0.007, *P*[IL-1β + TNF-α(7 days) versus Ctr(7 days)] = not significant, *P*[Ctr(14 days) versus IL-1β + TNF-α(withdrawal at 14 days)] < 0.0007; fig. S8, B and C}. These findings indicate that varicose projection astrocytes are a transient phenomenon, appearing only under sustained neuroinflammatory conditions. This reversibility highlights the dynamic nature of varicose projection astrocytes, suggesting that interventions targeting inflammation could mitigate their formation.

### Varicose projection astrocytes are induced by pro-inflammatory stimuli in cerebral organoids

**Fig. 3.**
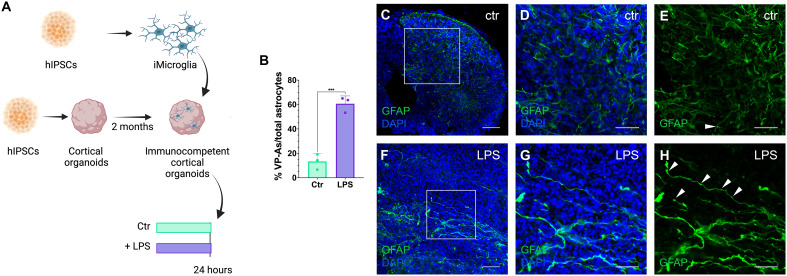
Varicose projection astrocytes are induced by LPS in human cortical organoids. (**A**) Schematic representation of the experimental timeline, created with BioRender [Created in BioRender. Falcone, C. (2026) https://BioRender.com/8v2meq6]. hiPSCs were differentiated into cortical organoids for 2 months and then injected with induced microglia (iMicroglia) and cultured for an additional 2 months to generate immunocompetent cortical organoids. Organoids were treated with LPS for 24 hours. (**B**) Quantification of varicose projection astrocytes as a percentage of total GFAP^+^ astrocytes in control (Ctr) and LPS-treated conditions, showing a significant increase in varicose projection astrocytes following LPS exposure. Data are presented as means ± SEM; statistical significance was assessed by the two-tailed *t* test (*n* = 3, **P < 0.001). (**C** to **H**) Representative IF images of GFAP^+^ astrocytes (green) and DAPI-stained nuclei (blue) in cortical organoids under control (C to E) and LPS-treated (F to H) conditions. (D and E) Higher magnification of GFAP^+^ astrocytes in control organoids, showing typical astrocyte morphology. (G and H) Higher magnification of GFAP^+^ astrocytes in LPS-treated organoids, with arrowheads indicating the presence of varicose projection astrocytes. Scale bars, 100 μm [(C) and (F)] and 50 μm [(D), (E), (G), and (H)]. VP-As, varicose projection astrocytes.

To further investigate the link between pro-inflammatory stimulation and varicose projection astrocytes in a complex three-dimensional (3D) human model, we examined their presence in immunocompetent cerebral organoids (i.e., organoids with microglia). Cortical organoids were generated using an optimized protocol (*25*). We differentiated organoids under gentle shaking for 4 months and added iPSC-derived microglia to 2-month-old organoids (Fig. 3A). To assess whether varicose projection astrocytes could be induced by inflammatory stimuli, we treated 4-month-old organoids with LPS (10 ng/ml) for 24 hours and performed GFAP IF staining. Quantification revealed a significant increase in varicose projection astrocyte density in LPS-treated organoids compared to controls (% varicose projection astrocytes/total GFAP^+^ astrocytes: Ctr = 13.39 ± 3.65%; LPS = 60.66 ± 4.21%, *P* < 0.0001; Fig. 3, B to H). We note that inflammatory stimulation induces a general increase in GFAP signal intensity and process complexity in organoid astrocytes, consistent with a reactive astrocytic response. However, varicose projection astrocytes were identified only when astrocytic processes displayed clearly discrete, regularly spaced bead-like swellings that were morphologically distinct from generalized process thickening or diffuse “bumpy” appearance. Astrocytes exhibiting uniformly thickened or irregularly contoured processes without discrete varicosities were not classified as varicose projection astrocytes. These findings indicate that varicose projection astrocytes emerge in response to inflammatory stimulation within a physiologically relevant human 3D system, reinforcing their association with neuroinflammatory conditions.

### Varicosities contain markers of extracellular vesicles and subcellular organelles.

To elucidate the nature of the varicosities (the hallmark of varicose projection astrocytes), we investigated whether they contain markers for extracellular vesicle (EV) components or specific cellular organelles, such as mitochondria, Golgi complex and endoplasmic reticulum (ER). We performed a treatment of hiPSC-derived astrocytes with 100 ng/ml each of IL-1β and TNF-α for 7 days, followed by IF staining for various subcellular markers.

**Fig. 4.**
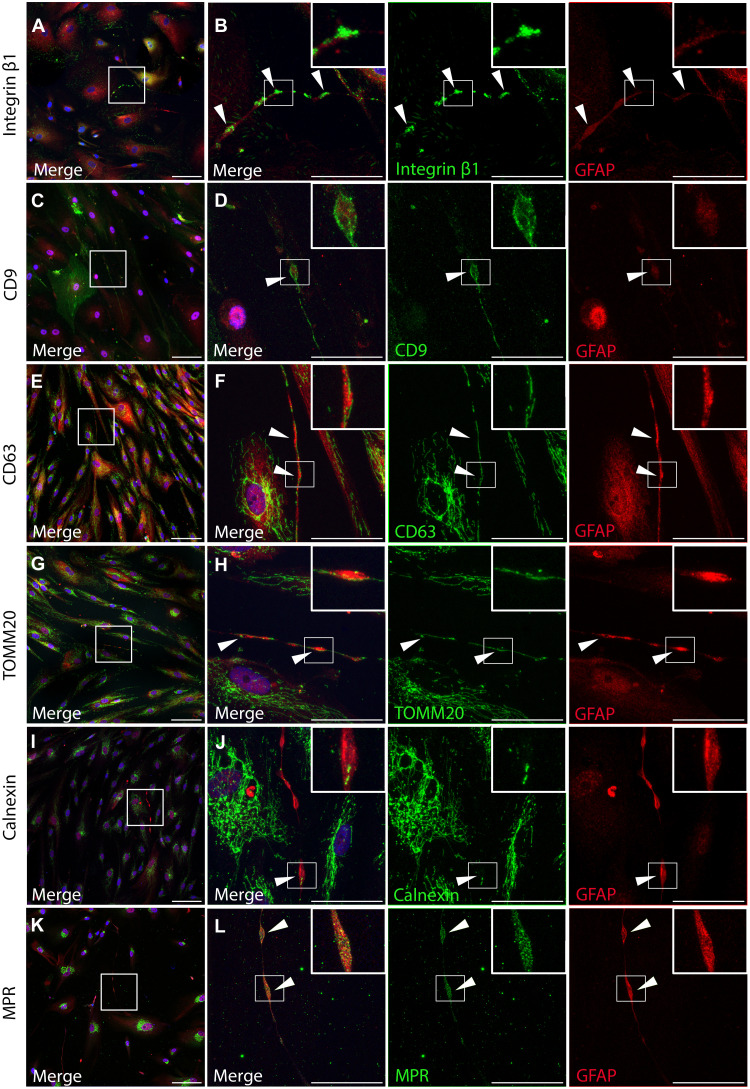
Varicosities contain EV markers and subcellular organelles. (**A** and **B**) Representative IF images of astrocytes stained for GFAP (red), integrin β1 (green), and DAPI (blue). Integrin β1 colocalizes with varicosities (arrowheads) (*n* = 3). (B) Higher magnification of the boxed region in (A), highlighting varicosities. (**C** and **D**) Representative IF images of astrocytes stained for GFAP (red), CD9 (green), and DAPI (blue). CD9 colocalizes with varicosities (arrowheads) (*n* = 3). (D) Higher magnification of the boxed region in (C), highlighting varicosities. (**E** and **F**) Representative IF images of astrocytes stained for GFAP (red), CD63 (green), and DAPI (blue). CD63 colocalizes with varicosities (arrowheads) (*n* = 3). (F) Higher magnification of the boxed region in (E), highlighting varicosities. (**G** and **H**) Representative IF images of astrocytes stained for GFAP (red), TOMM20 (green), and DAPI (blue). TOMM20, a mitochondrial outer membrane protein, reveals fragmented mitochondria within varicosities (arrowheads) (*n* = 3). (H) Higher magnification of the boxed region in (G), highlighting varicosities. (**I** and **J**) Representative IF images of astrocytes stained for GFAP (red), calnexin (green), and DAPI (blue). Calnexin, an ER membrane protein involved in protein folding, is detected within varicosities (arrowheads) (*n* = 3). (J) Higher magnification of the boxed region in (I), highlighting varicosities. (**K** and **L**) Representative IF images of astrocytes stained for GFAP (red), MPR (green), and DAPI (blue). MPR, a marker of the TGN, is detected within varicosities (arrowheads) (*n* = 3). (L) Higher magnification of the boxed region in (K), highlighting varicosities. Scale bars, 100 μm [(A), (C), (E), (G), (I), and (K)] and 50 μm [(B), (D), (F), (H), (J), and (L)].

First, we stained astrocytes for GFAP (to label varicose projection astrocytes) and EV markers, including integrin β1, CD9, and CD63. Integrin β1, a multifunctional cell surface receptor involved in extracellular matrix interactions and EV uptake (*26*), was localized within the varicosities (Fig. 4, A and B). Similarly, tetraspanin family proteins cluster of differentiation 9 (CD9) and cluster of differentiation 63 (CD63), key components of EV membranes and involved in their biogenesis (*27*, *28*), colocalized with varicosities (Fig. 4, C and D and E and F, respectively).

Next, we assessed the presence of organelles. Staining for TOMM20, a mitochondrial outer membrane protein (*29*), revealed portions of mitochondria within varicosities (Fig. 4, G and H), possibly indicative of a role in metabolic regulation during inflammation. Calnexin, an ER membrane protein involved in protein folding and quality control (*30*), was also present in varicose projection astrocytes, as evidenced by colocalization with GFAP at the level of varicosities (Fig. 4, I and J). Last, we examined the mannose 6-phosphate receptor (MPR), a marker for the trans-Golgi network (TGN) (*31*), and identified its presence in varicosities (Fig. 4, K and L).

Although all tested markers (including CD9, CD63, and integrin β1) were detected within varicosities, their distribution along the astrocytic processes was not homogeneous. Notably, integrin β1 showed a pronounced enrichment within varicosities, with only faint signal along the intervening segments, suggesting compartmentalized localization of specific proteins within these structures.

This characterization demonstrates that varicose projection astrocytes harbor a range of EV markers and intracellular organelles, suggesting their potential role in cellular communication and stress responses under inflammatory conditions.

### Varicose projection astrocytes are induced by pro-inflammatory conditions in mouse astrocytes both in vitro and in vivo

**Fig. 5.**
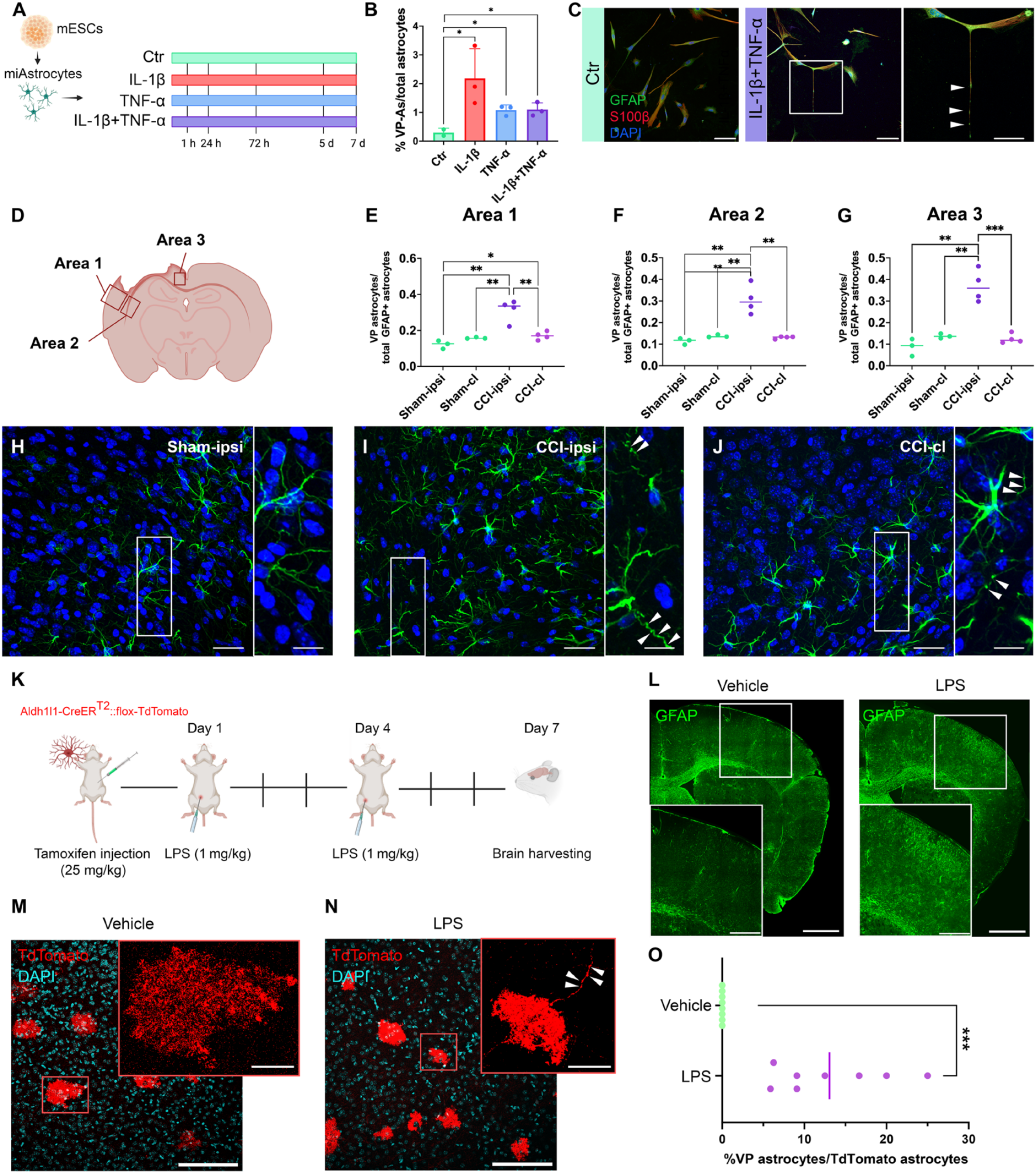
Varicose projection astrocytes are induced by pathological conditions in the mouse both in vitro and in vivo. (**A**) Experimental design. mESC-derived astrocytes, miAstrocytes; h, hours; d, days. (**B**) Quantification of varicose projection astrocytes as a percentage of total astrocytes. Data are presented as means ± SEM; statistical significance was assessed by the one-way ANOVA with Tukey’s multiple comparisons test (*n* = 2, 3, 3, and 3; *P < 0.05, **P < 0.01, and ***P < 0.001). (**C**) Representative IF images of miAstrocytes stained for GFAP (green), S100β (red), and DAPI (blue) under control and IL-1β + TNF-α–treated conditions, with a higher magnification of the boxed regions (IL-1β + TNF-α–treated condition). (**D**) Schematic representation of the brain areas analyzed (Areas 1 to 3), created with BioRender [Created in BioRender. Falcone, C. (2026) https://BioRender.com/hyecb8k]. CCI, controlled cortical impact. (**E** to **G**) Quantification of varicose projection astrocyte density (as a percentage of total GFAP^+^ astrocytes) in Area 1 (E), Area 2 (F), and Area 3 (G). Data are means ± SEM; unpaired two-tailed *t* tests (sham, CCI *n* = 3 and 4; *P < 0.05, **P < 0.01, and ***P < 0.001). (**H** to **J**) Representative GFAP images in sham-ipsilateral (H), CCI-ipsilateral (I), and CCI-contralateral (J). (**K**) Experimental design of the LPS inflammation model, created with BioRender [Created in BioRender. Falcone, C. (2026) https://BioRender.com/q9lbek3]. (**l**) Representative images of GFAP immunostaining (green) in vehicle and LPS-treated mouse brains showing increased astrocyte reactivity under the LPS condition. (**M** and **N**) Representative images of sparsely TdTomato-labeled astrocytes (red) from vehicle (M) and LPS-treated (N) mice. (**O**) Quantification of varicose projection astrocytes as a percentage of TdTomato+ astrocytes in vehicle versus LPS-treated mice. Data are presented as means ± SEM; unpaired two-tailed Student’s *t* test (*n* = 8 and 8; ***P < 0.001). Scale bars, 50 μm [(C), (H), (I), and (J)], 25 μm [(C), (H), (I), (J), (M), and (N) indentations], 1 mm (L), 500 μm [(L) indentations], and 100 μm [(M) and (N)]. Arrowheads point to varicosities.

To determine whether varicose projection astrocytes are an inflammation-associated feature, we conducted a similar in vitro experiment using mouse embryonic stem cell (mESC)–derived astrocytes (Fig. 5A). First, we confirmed the astrocytic identity of the mouse cells by immunostaining for established markers, including GFAP, S100b, Aldh1l1, Kir4.1, EAAT1, GS, Sox9, and vimentin (figs. S9 and S10).

Next, we exposed the mESC-derived mouse astrocytes to IL-1β, TNF-α, or a combination of the two cytokines, each at a concentration of 100 ng/ml, following published protocols (*32*–*34*) (Fig. 5A). After 7 days of stimulation, we observed an increase in varicose projection astrocytes in mouse astrocyte cultures under all three different conditions of stimulation, marking the identification of varicose projection astrocytes in this species [Fig. 5B, % varicose projection astrocytes/total astrocytes in culture: Ctr = 0.60 ± 0.11%, IL-1β = 2.18 ± 0.59%, TNF-α = 1.08 ± 0.10%, IL-1β + TNF-α = 1.10 ± 0.14%, with *P*(Ctr versus IL-1β) < 0.05, *P*(Ctr versus TNF-α) < 0.007, *P*(Ctr versus IL-1β + TNF-α) < 0.01]. As indicated by white arrowheads (Fig. 5C), varicose projection astrocytes with structural characteristics similar to human varicose projection astrocytes were present across all three treatment conditions.

To rule out potential artifacts arising from in vitro conditions, we conducted two complementary in vivo experiments in mice. First, we used a controlled cortical impact (CCI) model, in which a single cortical injury was induced using a 1.1-mm stainless steel flat-tip impactor 11-week-old mice, as previously described (*35*). Twenty-four hours after the impact, we euthanized the animals, fixed the brains, and immunostained the sections for GFAP. We then quantified the density of varicose projection astrocytes in CCI versus sham animals across three cortical regions (Areas 1 to 3; Fig. 5D). In all three areas, we observed a significant increase in varicose projection astrocyte density in the CCI-ipsilateral hemisphere compared with shams [% varicose projection astrocytes/total GFAP^+^ astrocytes: (Area 1; Fig. 5E) sham-ipsi = 12.15 ± 1.3%, sham-cl = 15.79 ± 0.22%, TBI-ipsi = 31.24 ± 3.07%, TBI-cl = 17.0 ± 1.12%, with *P*(sham-ipsi versus TBI-ipsi) < 0.002, *P*(sham-ipsi versus TBI-cl) < 0.02, *P*(TBI-ipsi versus TBI-cl) < 0.002; (Area 2; Fig. 5F) sham-ipsi = 11.39 ± 0.86%, sham-cl = 13.72 ± 0.36%, TBI-ipsi = 30.60 ± 3.33%, TBI-cl = 13.09 ± 0.27%, with *P*(sham-ipsi versus TBI-ipsi) < 0.002; (Area 3; Fig. 5F) sham-ipsi = 8.72 ± 2.33%, sham-cl = 13.75 ± 0.62%, TBI-ipsi = 36.96 ± 3.7%, TBI-cl = 12.53 ± 1.12%, with *P*(sham-ipsi versus TBI-ipsi) < 0.001]. Although Areas 2 and 3 showed no significant changes contralaterally, Area 1 (closer to the pia) displayed a modest but significant increase in the CCI-contralateral hemisphere compared to shams (Fig. 5, E to J).

To further corroborate the increase in varicose projection astrocyte density in mice under pathological conditions, we performed a second in vivo experiment. We used a transgenic line expressing a tamoxifen-dependent Cre recombinase driven by the astrocyte-specific Aldh1l1 promoter, along with a Cre-dependent TdTomato reporter (Aldh1l1CreERt2::floxTdTomato), enabling sparse astrocyte labeling after tamoxifen administration. We administered tamoxifen (25 mg/kg) on D0. Then, we intraperitoneally injected the mice with LPS (1 mg/kg) on D1 and D4. Control animals received injections of 1× phosphate-buffered saline (PBS). On D7, we euthanized the animals and fixed their brains (Fig. 5K). Sections were immunostained for GFAP and red fluorescent protein (RFP) (to enhance the tdTomato signal and therefore visualize sparsely labeled astrocytes). We found a significant increase in varicose projection astrocyte density in the primary motor cortex of LPS-treated mice compared to control mice [% varicose projection astrocytes/total TdTomato+ astrocytes: vehicle (control) = 0%, LPS-treated = 13.05 ± 2.44%, with *P* < 0.0001; Fig. 5, L to O, and fig. S11].

These findings support the hypothesis that varicose projection astrocytes are not a specific astrocyte subtype but rather a reactive phenotype induced by pro-inflammatory stimulation. This also suggests that varicose projection astrocyte formation may represent a conserved astrocytic response to pathology across diverse mammalian species, rather than being exclusive to primates.

### Varicose projection astrocyte density increases in patients with neurodegenerative diseases and epilepsy

**Fig. 6.**
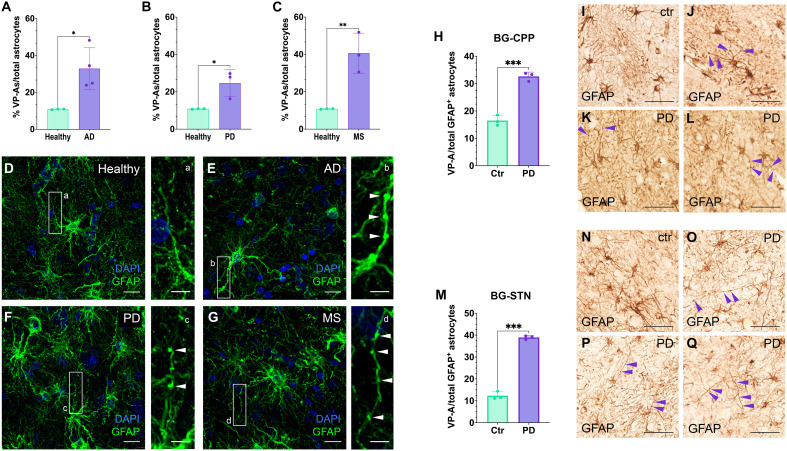
Varicose projection astrocyte density increases in human neurodegenerative diseases. (**A** to **C**) Quantification of varicose projection astrocytes as a percentage of total astrocytes in the prefrontal cortex of brains from (A) AD, (B) PD, and (C) MS, compared to controls. Data are presented as means ± SEM; two-tailed *t* test (*n* = 3; *P < 0.05, **P < 0.01). (**D** to **G**) Representative IF images of GFAP^+^ astrocytes (green) and DAPI nuclei (blue) in healthy (D) and diseased (E) AD, (F) PD, and (G) MS conditions. (a to d) Higher magnification of the boxed regions in (D), (E), (F), and (G), respectively. Scale bars, 30 μm. (**H** and **M**) Quantification of varicose projection astrocytes as a percentage of total GFAP^+^ astrocytes in the (H) BG-CPP and (M) BG-STN of PD and healthy controls (Ctr). Data are presented as means ± SEM; two-tailed *t* test (*n* = 3; ***P < 0.001). (**I** to **Q**) Representative images of GFAP^+^ astrocytes in control (I and N) and PD [(J) to (L) and (N) to (Q)] samples from the BG-CPP [(J) to (L)] and BG-STN [(O) to (Q)]. Scale bars, 50 μm.

If varicose projection astrocytes accumulate under pathological conditions, particularly in the presence of heightened neuroinflammation, their density might be increased in human neurological diseases. To investigate this, we examined postmortem human prefrontal cortex samples from patients with neurodegenerative disorders known for their inflammatory components (*36*), including AD, PD, and MS, and compared them to control samples from individuals without neurological disorders. IF staining for GFAP revealed a significant increase in varicose projection astrocyte density in all three diseases. Specifically, when we calculated the density of varicose projection astrocytes as GFAP^+^ varicose projection astrocytes/total no. of GFAP^+^ astrocytes, AD showed a 2.6-fold increase in varicose projection astrocyte density (AD analysis: Ctr = 10.85 ± 0.13%, AD = 27.79 ± 5.63%, *P* < 0.01; Fig. 6, A, D, and E), PD showed a 2.3-fold increase (PD analysis: Ctr = 10.85 ± 0.13%, PD = 24.62 ± 4.22%, *P* < 0.01; Fig. 6, B and F), and MS showed a 3.7-fold increase (MS analysis: Ctr = 10.85 ± 0.13%, MS = 40.69 ± 6.18%, *P* < 0.004; Fig. 6, C and G), in respect to healthy controls. Incidentally, we also observed varicosities in interlaminar astrocytes (ILAs) in pathological samples across all three diseases. Notably, control specimens contained some (but much fewer) varicose projection astrocytes, likely due to the advanced age of the specimens.

In the samples from disease advanced stages, varicose projection astrocytes were localized in all cortical layers and not only in the deepest ones, suggesting that this morphological change is a common astrocytic reaction and not something related to a single subpopulation of astrocytes. When the neurodegenerative disorder is at an advanced state, varicosities increase in number and size, consistent with the stressed state of the astrocytes (Fig. 6, E to G). We found that varicose projection astrocytes are also detectable by using another, well-known astrocyte marker S100β (fig. S12).

To assess whether varicose projection astrocytes are restricted to the cerebral cortex, we examined subcortical regions of postmortem PD brains, specifically the basal ganglia (BG) including the caudate nucleus, putamen, and globus pallidus (BG-CPP) and subthalamic nucleus (BG-STN). Varicose projection astrocytes were present in these regions, with significantly increased densities in PD in both BG-CPP and BG-STN. Specifically we found an ∼2-fold increase in varicose projection astrocyte density in the BG-CPP (density of varicose projection astrocytes in the BG-CPP calculated as GFAP^+^ varicose projection astrocyte/total no. of GFAP^+^ astrocytes: Ctr = 16.48 ± 1.11%, PD = 32.62 ± 0.92%, *P* < 0.0002; Fig. 6, H to L) and an ∼3-fold increase in the BG-STN (density of varicose projection astrocytes in the BG-STN calculated as GFAP^+^ varicose projection astrocytes/total no. of GFAP^+^ astrocytes: Ctr = 12.35 ± 1%, PD = 38.96 ± 0.5%, *P* < 1.06 × 10^−5^; Fig. 6, M to Q).

**Fig. 7.**
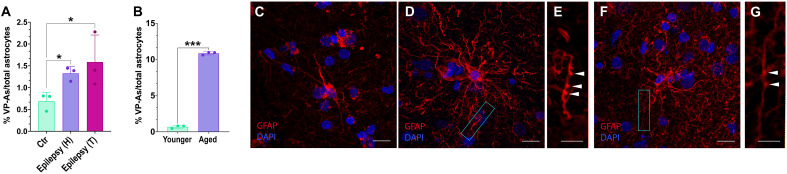
Varicose projection astrocyte density increases in epilepsy and with aging. (**A**) Quantification of varicose projection astrocytes in surgical resections from patients with hippocampal sclerosis [epilepsy (H)] and tumor-related epilepsy [epilepsy (T)]. Data are means ± SEM; two-tailed *t* test (*n* = 3; *P < 0.05). (**B**) Quantification of varicose projection astrocytes in young brains versus aged brains. Data are means ± SEM; two-tailed *t* test (*n* = 3; ***P < 0.001). (**C** to **G**) Representative IF images of GFAP^+^ astrocytes (red) and DAPI-stained nuclei (blue) in cortical tissue from controls (C), [epilepsy (H)] (D and E) and [epilepsy (T)] (F and G). (E and G) Higher magnification of boxed regions in (D) and (F), respectively. Scale bars, 50 μm [(C), (D), and (F)] and 25 μm [(E) and (G)]. Arrowheads point to varicosities.

We also investigated varicose projection astrocyte density in surgical resection from patients with epilepsy caused by hippocampal sclerosis or brain tumors. Varicose projection astrocytes were significantly more frequent in patients with epilepsy due to hippocampal sclerosis, showing an ∼2-fold increase in density (density of varicose projection astrocytes calculated as GFAP^+^ varicose projection astrocytes/total no. of GFAP^+^ astrocytes: Ctr = 0.69 ± 0.14%, hippocampal sclerosis–epilepsy = 1.33 ± 0.09%, *P* < 0.01; Fig. 7, A and C to E); patients with epilepsy due to brain tumors showed a similar density increase (density of varicose projection astrocytes calculated as GFAP^+^ varicose projection astrocytes/total no. of GFAP^+^ astrocytes: Ctr = 0.69 ± 0.14%, tumor-associated epilepsy = 1.59 ± 0.36%, *P* < 0.04; Fig. 7, A, C, F, and G).

These findings underscore that varicose projection astrocytes represent a hallmark of a subpopulation of reactive astrocytes under neuropathological conditions, with widespread occurrence across cortical and subcortical regions in both neurodegenerative and epileptic pathologies.

### Varicose projection astrocytes accumulate with age

Previous studies have shown that varicose projection astrocytes were not detected in neonatal or infant human brains (*21*, *22*), suggesting that their presence may be associated with aging or pathological conditions. Given that aging is linked to the accumulation of various brain stressors and pathological burden, we investigated whether varicose projection astrocyte density increases over time. To test this, we compared control brain tissues obtained from surgical resections of mid-age patients (age range = from 31 to 61 years old) with human postmortem brain samples from aged human controls (without diagnosed neurological disorders at the moment of death; age range = 77 to 81 years old). Our analysis revealed a significant increase in varicose projection astrocytes in older brains compared to younger ones, further supporting the idea that varicose projection astrocytes may emerge in response to accumulated cellular stress or pathological processes associated with aging (density of varicose projection astrocytes calculated as GFAP^+^ varicose projection astrocytes/total no. of GFAP^+^ astrocytes: younger = 0.69 ± 0.14%, aged = 10.85 ± 0.16%, *P* < 10 × 10^−7^; Fig. 7B).

## DISCUSSION

This study changes our understanding of varicose projection astrocytes by establishing their presence in mice and tigers and their link to neuropathology and neuroinflammation, challenging the traditional view of varicosities as mere structural features of certain astrocytic subtype. Our findings suggest that varicose projection astrocytes are not a physiological but rather a reactive phenotype induced under specific pathological conditions. By using multiple human-based models, including pure and mixed hiPSC-derived astrocyte cultures, pure hNSC-derived astrocyte cultures, 3D cortical organoids, human postmortem samples, and surgical resections, we provide robust evidence of their emergence under pathological conditions. This has significant implications for human neuropathology, given the species-specific differences in astrocyte properties and the unique features of human diseases (*18*, *36*–*39*).

Our approach accounted for established pathways of astrocyte-microglia cross-talk (*8*, *9*, *15*, *40*), using direct cytokine stimulation (TNF-α and IL-1β) in 2D cultures lacking microglia and LPS-induced microglial activation in organoids (*41*), thus demonstrating that varicose projection astrocytes arise from both direct and indirect inflammatory signaling. Identifying varicose projection astrocytes across multiple human pathologies underscores the potential of astrocyte varicosities as biomarkers for neuropathological process, adding to previously used canonical markers such as GFAP up-regulation and morphological hypertrophy. This raises intriguing questions about the molecular mechanisms that distinguish varicose projection astrocytes from other reactive astrocytes and whether varicose projection astrocytes represent a specific astrocytic program or a pathway within the continuum of reactive states. Moreover, the increased presence of varicose projection astrocytes in subcortical regions such as BG in PD (areas relevant to PD pathology) represents an intriguing finding, suggesting that these astrocytes respond adaptively according to regional vulnerability in the brain. Their presence across multiple cortical layers and subcortical regions suggests that varicose projection astrocytes might play a role in propagating inflammation or exacerbating neuronal dysfunction. However, we cannot exclude a potential neuroprotective role. The overlap of varicosities between varicose projection and ILAs in pathological states also warrants further investigation as it hints at shared mechanisms or functions (*21*). In addition, the observed increase in varicose projection astrocytes in aged human brains compared to younger samples suggests that their accumulation may be linked to age-related brain changes, potentially driven by chronic cellular stress or the gradual onset of pathological processes. This finding aligns with the idea that varicose projection astrocytes are not a constitutive astrocyte subtype but rather emerge in response to accumulated physiological or pathological challenges over the life span, reinforcing their potential role as markers of pathological risk.

Furthermore, we provide the characterization of varicosities. We revealed the presence of EV markers, components of mitochondria, Golgi complex, and ER within varicosities, indicating potential roles in cellular communication, metabolic stress management, and intercellular signaling under pathological or inflammatory conditions, as previously reported for reactive astrocytes (*26*, *42*, *43*). Given this composition, one intriguing possibility is that the elongated varicose processes enable long-distance delivery of metabolic substrates or signaling vesicles across brain regions. Another possibility is that the evenly spaced varicosities could act as relay points for Ca^2+^ or other intracellular signals, allowing varicose projection astrocytes to propagate information or stress responses over extended spatial domains. Although speculative, these features suggest that the distinct morphology of varicose projection astrocytes may be linked to specialized functional roles that differ from protoplasmic astrocytes. It is also possible that varicose projection astrocytes represent a pathology-driven astrocytic program distinct from traditional reactive states, in which inflammatory cues induce certain astrocytes toward developing varicosities rather than or in addition to the canonical hypertrophy. The fact that they show a broader distribution across cortical layers in advanced diseases (rather than being restricted to deep cortical layers) further raises the possibility that they either differentiate locally under pathological cues or migrate across brain regions as pathology progresses. In addition, the enrichment of integrin β1 within varicosities is intriguing and suggests potential molecular specialization: Validating integrin β1 as a specific in vivo marker of varicose projection astrocytes will be the focus of dedicated future studies and is beyond the scope of the present work.

We acknowledge that in vitro systems can produce nonphysiological morphologies, and we addressed this by (i) validating the expression of other astrocyte markers in the varicose projection astrocytes themselves, such as SOX9, ALDH1L1, GLAST, and Cd49f, and (ii) confirming varicose projection astrocyte features in two independent in vivo mouse models. Accordingly, our operational definition of varicose projection astrocytes prioritized the presence of varicosities rather than the absolute process length, recognizing that extreme elongation is context dependent and often attenuated in vitro. In dense 3D systems such as organoids, inflammatory activation can produce globally elongated and thickened astrocytic processes; for this reason, elongation and general process “bumpiness” were not used as defining features. Instead, classification relied on the presence of discrete, regularly spaced varicosities, a feature that was absent in most reactive astrocytes and selectively enriched following inflammatory stimulation. The biological relevance of this definition is supported by our in vivo mouse models, where varicose astrocytic processes emerge under inflammatory and traumatic conditions and frequently display pronounced elongation. Using both a CCI paradigm and LPS-induced neuroinflammation in Aldh1l1CreERt2::floxTdTomato transgenic mice, we observed a significant increase in the density of varicose projection astrocytes compared to controls, including within sparsely labeled astrocyte populations independent of GFAP expression/staining. These results demonstrate that varicose projection-like morphologies emerge in vivo under inflammatory and traumatic conditions, supporting their biological relevance rather than a culture-induced artifacts. Moreover, our finding that varicose projection astrocytes are not only restricted to hominoids (*17*, *21*) but also appear in other mammals, like mice and tigers, challenges the prevailing view of their hominoid-specific nature. Varicose projection astrocyte formation may be therefore a conserved astrocytic response to neuropathology, underscoring their broader biological significance across species. This also raises important questions about the evolutionary pressures that shaped the reactive capabilities of astrocytes and whether similar structures exist in other vertebrates. One might question why varicose projection astrocytes have not previously been described in other species. However, varicosities have been reported in ferret astrocytes (*23*), in radial glial cells of early postnatal cats (*44*), and in neonatal pigs under hypoxia (*45*). These earlier observations did not explicitly classify these structures as varicose projection astrocytes. One explanation for their apparent scarcity in nonhominoid species could be that varicose projection astrocytes accumulate progressively with aging, consistent with our hypothesis of accumulated lifelong pathological burden.

Although early descriptions of varicose projection astrocytes emphasized markedly elongated projections, our data indicate that varicosities can also occur on shorter astrocytic processes, both in vitro and in vivo, including in human pathological samples. This observation supports the use of an operational definition centered on the presence of discrete, regularly spaced varicosities rather than absolute process length, and suggests that varicosity formation represents a context-dependent astrocytic response rather than a fixed morphological trait.

Future work should focus on elucidating molecular mechanisms that regulate the formation of varicose projection astrocyte formation, including pathways such as NF-κB (*9*, *46*) and STAT3 (signal transducer and activator of transcription 3) signaling (*5*, *14*, *47*–*50*), the role of these astrocytes in neuropathology, and whether they exacerbate neuronal damage or exert neuroprotective functions. The observed reversibility of varicose projection astrocyte formation upon cytokine withdrawal indicates potential therapeutic avenues for modulating their emergence and pathological effects. The fact that varicose projection astrocytes emerge in subcortical regions as well suggest that future studies should focus also on regional heterogeneity in varicose projection astrocyte formation and its implications for disease-specific astrocytic malfunctions. Last, the potential interplay between varicose projection astrocytes and other glial cells, such as other astrocytes, microglia, and oligodendrocytes, merits exploration as glial cross-talk is increasingly recognized as a key driver of neuropathology. Furthermore, we recognize that increases in varicose projection astrocyte density could theoretically result from enhanced GFAP visibility in reactive astrocytes. However, our observations of varicose projection morphology in tdTomato-labeled astrocytes—where labeling is independent of GFAP expression—argue against this being a staining artifact. In future work, we aim to incorporate additional techniques, such as dye-filling and high-resolution imaging (e.g., EM reconstruction), to further validate varicose projection astrocyte morphology and ensure unbiased identification.

This work aligns with and extends recent studies emphasizing the heterogeneity of astrocytic responses in neuropathology (*5*, *14*). Emerging evidence suggests that reactive astrocytes are not monolithic but exist on a spectrum influenced by context, region, and stimuli. Varicose projection astrocytes may represent a unique node on this spectrum, characterized by their distinct morphology and molecular composition, and open previously unidentified avenues for translational studies in neuroinflammatory and neurodegenerative diseases. Another open question is whether varicose projection astrocytes should be considered a specialized subtype within the continuum of reactive states or a more specific trajectory that is mechanistically distinct from “classical” reactive astrocytes. Addressing these possibilities will refine our definition of varicose projection astrocytes and advance the broader framework of astrocyte heterogeneity in disease.

## MATERIALS AND METHODS

### hiPSC culture and differentiation into astrocytes

hiPSCs were derived from normal human skin fibroblasts through episomal reprogramming methods. The hiPSC line ASE-9209 (47-year-old female) was from Applied StemCell Inc. hiPSCs were cultured and expanded in mTeSR Plus (STEMCELL Technologies, 05825) on Matrigel (Corning, 354277)–coated dishes, changing the medium every other day. hiPSCs were split using 1 ml of ReLeSR (STEMCELL Technologies, 100-0484) for each well of the 6-well plate for 1 min. Then, 900 μl of ReLeSR was removed and cells were incubated for 7 min at 37°C. Dulbecco’s modified Eagle’s medium (DMEM; Thermo Fisher Scientific, 31966047) was added to detach hiPSCs and collect them in a tube. Cells were centrifuged, then the supernatant was removed and resuspended in 1 ml of mTeSR medium.

### Human astrocyte cultures

hiPSCs were differentiated into astrocytes adapting a previously published protocol (*51*). hiPSCs were split manually with ReLeSR (STEMCELL Technologies, 100-0484) to generate the embryoid bodies (≈D5 of differentiation). Embryoid bodies were grown in suspension culture on a 6-well plate in DMEM/F-12 (Thermo Fisher Scientific, 11330032), with 1× N2 supplement (Invitrogen, 17502048) and Noggin (40 ng/ml; PeproTech, #120-10C) for 3 to 5 days. The medium was changed daily. After 3 to 5 days, the embryoid bodies were plated on a Matrigel (Corning, 354277)–coated 6-well plate in neural rosettes medium with DMEM/F-12, 1× N2 supplement, and laminin (1 μg/ml; Invitrogen, 2317-015) to form neural rosettes (neural progenitor cells in the form of neural rosettes) in 5 to 7 days. The medium was changed every other day. To differentiate immature astrocytes, the neural rosettes were mechanically dissociated and plated on a Matrigel-coated 6-well plate in astrocyte differentiation medium, consisting of DMEM/F-12, 1× N2 supplement, 1× B27-RA supplement (Invitrogen, 12587010), BMP4 (10 ng/ml; PeproTech, 120-05ET), and FGF-basic (20 ng/ml; PeproTech, 100-18B). The medium was changed every other day.

hNSCs (Alstem, catalog no. hNSC1) were differentiated into astrocytes following the protocol described below. Briefly, hNSCs were cultured in DMEM/F-12 (Thermo Fisher Scientific, 11330032) supplemented with N2 (1×), B27 (1×), GlutaMAX (1×), penicillin-streptomycin (1×), basic fibroblast growth factor (bFGF) (20 ng/ml; PeproTech, 100-18B), and epidermal growth factor (EGF) (20 ng/ml) and maintained on poly-l-ornithine (PLO; 20 μg/ml; Sigma-Aldrich, P3655)–coated and laminin (1 μg/ml; Sigma-Aldrich, L2020)–coated plates. For astrocyte differentiation, hNSCs were dissociated with Accutase (Millipore, SCR005) and seeded at 15,000 cells per well in 24-well plates (600 μl per well) on PLO/laminin-coated coverslips. Cells were initially maintained in an astrocyte differentiation medium consisting of DMEM/F-12 supplemented with N2 (1×), B27 (1×), penicillin-streptomycin (1×), BMP4 (10 ng/ml; PeproTech, 120-05ET), and bFGF (20 ng/ml; PeproTech, 100-18B), followed by a transition to a different differentiation medium containing DMEM/F-12 supplemented with N2 (1×), GlutaMAX (1×), penicillin-streptomycin (1×), BMP4 (10 ng/ml), and ciliary neurotrophic factor (CNTF) (20 ng/ml), to induce further maturation of astrocytes. The medium was changed every other day using a 2/3 volume exchange, and cells were differentiated for ∼2 to 3 weeks before experimentation.

Following fixation, immunocytochemistry was performed using antibodies against GFAP (Sigma-Aldrich, AB5541), Sox9 (Sigma-Aldrich, AB5535-25UG), GLAST/EAAT1 (Synaptic Systems, 250113), CD49f (Thermo Fisher Scientific, 14-0495-82), and ALDH1L1 (Antibodies Inc., 75-164-020), with 4′,6-diamidino-2-phenylindole (DAPI) (Thermo Fisher Scientific, D3571) used for nuclear staining. Secondary antibodies were applied at 1:1000.

### Mixed culture model

hiPSCs were differentiated following a previously published protocol (*52*). The colonies of hiPSCs were plated at 1 × 10^5^ cells per well on a Matrigel-coated 6-well plate and fed daily with mTeSR. After 2 days, differentiation, to generate OLIG2^+^ progenitors, was induced by adding neural induction medium with retinoic acid (Sigma-Aldrich, R265), SB431542 (Sigma-Aldrich, 616461), and LDN193189 (Sigma-Aldrich; SML0559) until D8. On D8, the medium was switched to N2 medium until D12 with 1× N2 supplement (Invitrogen, 17502048), changing the medium daily. On D12, cells were enzymatically dissociated using Accutase (Thermo Fisher Scientific, A1110501), breaking the monolayer into small clumps and transferring them into an ultralow attachment 6-well plate. The N2B27 medium was added in each well with 1× N2 supplement and 1x B27 without vitamin A (Invitrogen, 12587010). Two-thirds medium change was performed every other day by transferring aggregates. On D20, the medium was switched to platelet-derived growth factor (PDGF)–enriched medium using two-thirds medium change. These steps were repeated until D30 to obtain the aggregation of OLIG2^+^ cells in spheres. On D30, the round aggregates, with a diameter between 300 and 800 μm and with a gold or brown center, were picked with a p200 pipette and plated on a 6-well plate coated with PLO (Sigma-Aldrich, P4957-50ML) and laminin (Sigma-Aldrich, L2020-1MG) (20 spheres per well) and the medium was switched to glia medium. The medium was changed every other day, replenishing two-thirds of the medium with a fresh one until D55. In this culture, neurons and astrocytes are mostly present, which can be maintained and expanded on a multiwell plate coated with PLO and laminin in glial medium.

### Differentiation of mESCs into astrocytes

The mESCs were differentiated following a previously published protocol (*53*). The mESCs were dissociated using Accutase (Thermo Fisher Scientific, A1110501) and cultured on Matrigel (Corning, 354277) in ESCs medium before induction. On D1, the medium was replaced by NE medium 1 comprising DMEM/F-12 supplemented with 1× B27 (Invitrogen, 12587010), 1× N2 (Invitrogen, 17502048), 1× NEAA (nonessential amino acids, Euroclone, ECB3054D), 2-mercaptoethanol (0.1 mM, Sigma-Aldrich, M7154), Noggin (100 ng/ml; PeproTech, #120-10C), SB431542 (20 μM; Sigma-Aldrich; 616461), dorsomorphin (2 μM; Sigma-Aldrich, P5499-5MG), and CHIR99021 (3 μM; Sigma-Aldrich, SML1046-5MG). On D4, neural rosettes–like colonies were dissociated with Accutase, and the medium was replaced with NE medium 2 consisting of DMEM/F-12 supplemented with 1× N2, FGF2 (20 ng/ml; PeproTech/ImmunoTools, 100-18B), and glucose (1.6 g/liter; Sigma-Aldrich, G8270). The medium was maintained for an additional 4 days in ultralow attachment plates to obtain neurospheres. On D8, neurospheres were collected, dissociated with Accutase, and transferred to Matrigel-coated plates in NE medium 3 with DMEM/F-12, supplemented with 1× N2, 1× B27, insulin (20 μg/ml; Sigma-Aldrich, 19278), glucose (1.6 g/liter; Sigma-Aldrich, G8270), EGF (20 ng/ml; ImmunoTools, 11343406), and FGF2 (20 ng/ml; PeproTech/ImmunoTools, 100-18B). After 1 day, almost all cells dissociated from neurospheres being attached to the plate as NESCs, subsequently cultured in the NE medium 3 to achieve cell expansion and differentiation in neurons and oligodendrocyte precursor cells (OPCs). On D9, ESC-derived NESCs were dissociated using Accutase and plated on a 6-well plate coated with Matrigel. Cells were grown for 5 days in OPC differentiation media with DMEM/F-12, 1× B27, 1× N2, smoothened agonist (SAG) supplements (0.4 μM; Sigma-Aldrich, 566661-500UG), FGF2 (20 ng/ml; PeproTech/ImmunoTools, 100-18B), and platelet-derived growth factor-AA (PDGF-AA, 20 ng/ml; ImmunoTools, 11343683). The resulting OPCs, grown and expanded in OPC media, are ready to be differentiated into astrocytes. All media were replaced every 2 days. Astrocytes were induced with Astro medium for 7 days with DMEM/F-12, supplemented with 1× B27, 1× N2, and BMP4 (20 ng/ml; PeproTech/ImmunoTools).

### Cytokine treatments

The pure cultures of astrocytes derived from hiPSCs were plated on a 12-multiwell plate and treated with IL-1β (100 ng/ml; ImmunoTools, 11340012), TNF-α (100 ng/ml; PeproTech, 300-01A), or a combination of IL-1β (100 ng/ml) and TNF-α (100 ng/ml). Controls were treated with 1× PBS (pH 7.4) (Thermo Fisher Scientific, 10010023). The starting concentration of 100 ng/ml was chosen on the basis of published protocols (*32*–*34*). Astrocytes were treated for 1, 24, and 72 hours and 5 and 7 days. The medium was not replaced throughout the treatments.

The pure cultures of astrocytes derived from hNSCs were subjected to a full media exchange and treated with IL-1β and TNF-α (100 ng/ml each) for 7 days, with treatment media refreshed every other day. Two independent experimental cohorts were performed in parallel to assess astrocyte identity using additional markers (see the “Immunofluorescence” section).

The mixed culture was plated on a 12-well plate and treated with four different combined concentrations of cytokines for 7 days: (i) IL-1β (1 ng/ml) and TNF-α (1 ng/ml), (ii) IL-1β (10 ng/ml) and TNF-α (10 ng/ml), (iii) IL-1β (30 ng/ml) and TNF-α (30 ng/ml), and (iv) IL-1β (100 ng/ml) and TNF-α (100 ng/ml). 1× PBS (pH 7.4) was used as a treatment control. The medium was not replaced throughout the treatments.

Mouse astrocytes were plated on a 12-well plate and treated with IL-1β (100 ng/ml), TNF-α (100 ng/ml), or a combination of IL-1β (100 ng/ml) and TNF-α (100 ng/ml). 1× PBS (pH 7.4) (Thermo Fisher Scientific, 10010023) was used as a treatment control. The medium was not replaced throughout the treatments.

### Mice

Animal experiments for the CCI experiment were done at the Oxford University and approved by a local ethical review committee (AWERB) and conducted under the UK Animals (Scientific Procedures) Act 1986 (ASPA), under valid personal and project licenses (establishment local license: XEC303F12; project license: PPL3543271). CD-1 mice (*Mus musculus*) were acquired from Charles River and housed in individually ventilated cages on a 12-hour light/12-hour dark cycle, with ad libitum access to water and food. Cages were environmentally enriched with an activity wheel and nesting material. Male and female mice (11 weeks of age) were used for the CCI experiment.

Animal experiments for the LPS injection experiment were done at the Cajal Institute (Madrid). Procedures for care, welfare, and proper use of all experimental animals followed the European Parliament and Council Directive (2010/63/EU) and the Spanish regulation (R.D. 53/2013 and Ley 6/2013, 11 June) and were approved by our Institutional Animal Care and Use Committee (Comité de Ética de Experimentación Animal del Instituto Cajal) and by the Consejería del Medio Ambiente y Territorio (Comunidad de Madrid), with the assigned animal protocol number PROEX 294.8/23. For this experiment, a transgenic mouse line (*M. musculus*) expressing a tamoxifen-dependent Cre recombinase driven by the astrocyte-specific Aldh1l1 promoter, along with a Cre-dependent TdTomato reporter (Aldh1l1CreERt2::floxTdTomato), was used. A total of six animals (four females and two males) were included. Two additional males were processed but excluded due to the difficulty in locating the target cells in the sections. Animals were divided into two groups: LPS-treated and control.

### CCI model

Animals were anesthetized with isoflurane and placed in a stereotactic frame (Stoelting Co., USA). Following midline skin incision, a 2-mm craniotomy was performed over the somatosensory cortex. A CCI model was applied, as previously described (*35*). A single trauma was caused by a 1.1-mm stainless steel flat-tip impactor, at a speed of 1.5 m/s and a depth of 1 mm from the cortical surface. The bone flap was replaced and sealed with glue. The skin was sutured and reinforced with additional glue. Animals were allowed to recover in a heated chamber before being returned to their home cage. Sham animals underwent the same surgical procedure, including craniotomy, but without impact. After 24 hours, animals were perfusion fixed following terminal anesthesia, and brains were collected.

### LPS injection

Tamoxifen (25 mg/kg) was administered on D0. The next day, LPS (1 mg/kg) injections were performed on D1 and D4 using LPS (250 μg/ml) in 1× PBS. Control animals received injections of 1× PBS. On D7, animals were deeply anesthetized and transcardially perfused with 30 ml of 4% paraformaldehyde (PFA).

### Human and other animal tissues

The postmortem human samples were obtained from Netherlands Brain Bank. These specimens were fixed in formalin and paraffin embedded. The prefrontal cortex was selected for the analysis. More detailed information regarding the human donors is provided in table S1. The tissues were de-deparaffinized and embedded in optimal cutting temperature (OCT) (Bio-Optica Milano S.p.A., #059801), following the protocol published by Ciani *et al.* (*54*). Then, 40-μm-thick sections were cut using a cryostat (Microm HM550) and stained by IF (see below).

Postmortem paraffin-embedded brain samples included those from BG of patients with PD, divided into two blocks per each individual: One block included the BG-CPP and another block included the BG-STN (table S1). These samples were obtained from the Biobank “Biobanc-Hospital Clinic-IDIBAPS” in Barcelona, Spain.

The specimens of tigers (*P. tigris*) that died under human care were sent to the Department of Comparative Biomedicine and Food Science (BCA) at the University of Padua for routine postmortem examination. Brains were opportunistically gathered and, immediately following extraction, were preserved by immersion in 4% phosphate-buffered formalin. Following death, autolysis begins to decompose biological tissues, including the brain. Although, in laboratory settings, smaller animals can be perfused with fixative right after euthanasia, maintaining minimal postmortem intervals, larger animal brains collected during necropsies and strandings are stored in refrigerators at 4°C for several hours until the necropsy is conducted. This standard practice prevents damages caused by prolonged postmortem intervals, thereby preserving tissue quality and staining results. The prefrontal cortex was sampled on the basis of the study by Musil and Olson (*55*). We then embedded the tissues in OCT (Bio-Optica Milano S.p.A., #059801), as in the study by Ciani *et al.* (*54*), and cut 40-μm-thick sections at the cryostat (Microm HM550).

Permission to use human brain tissue (from surgical resections) was obtained from the Vilnius Regional Biomedical Research Ethics Committee (approval no. 2020/2-1202- 687). Human brain tissue specimens were obtained with the informed consent as requested by the Regional Ethics Committee (no. 2/2020 02 18). Human neocortical biopsy of tissue was sampled from either glioma tumor resection surgery (*n* = 3), using the distant cortex without tumor infiltration (*n* = 3), or access cortex tissue from epilepsy surgery (*n* = 3) obtained during the resection of epileptic foci. Detailed information regarding the donors is provided in table S1.

### Cortical organoids

Brain organoids were generated from the standardized human iPSC cell line KOLF2.1J (the Jackson Laboratory) using an optimized protocol published by Lancaster *et al.* (*25*). Briefly, each organoid was generated from 4000 iPSCs using AggreWell 800 (STEMCELL Technologies) using the commercial STEMdiff cerebral organoid kit (STEMCELL Technologies). Embryoid bodies generated were selected and, after 5 days in AggreWell, differentiated (for 5 days) and matured under gentle shaking (80 rpm in Minitron shaker from Infors HT) for up to 4 months. iPSC-derived microglia from KOLF2.1J were introduced in 2-month-old brain organoids. iPSCs were differentiated into hematopoietic progenitor cells (STEMdiff Hematopoietic Kit, STEMCELL Technologies) and differentiated 2 days in microglia before their inclusion by centrifugation (100*g*, 3 min) in brain organoids (STEMdiff Microglia Differentiation Kit, STEMCELL Technologies). Immunocompetent organoids generated were positive for the neuronal (SMI31 and MAP2), astrocytic (GFAP and S100b), oligodendrocytic (Sox10 and NogoA), and microglial (Iba1 and CD68) markers. GFAP staining (DAKO, 1:1000) was performed by IF in 4-month-old organoids after 24 hours of treatment with LPS (10 ng/ml).

### Tissue processing

Mouse brains from the CCI experiment were postfixed in 4% PFA for 24 hours at 4°C and cryoprotected in 30% sucrose for 48 hours. Brains were then frozen on dry ice and coronally sectioned using a Leica SM2000 R sliding microtome. Sections (30 μm) were stored in cryoprotectant solution at −20°C.

Mouse brain from the LPS injection experiment were postfixed in 4% PFA overnight at 4°C and then transferred to tris-buffered saline (TBS) and sectioned at 50 μm using a vibratome (Leica VT1000 S).

Postmortem tissues of the prefrontal cortex from humans and tigers were fixed in formalin for several days and subsequently incubated in 30% sucrose in PBS at 4°C for at least 2 days, allowing tissues to sink, which is essential for cryoprotection. After cryoprotection, tissues were embedded in OCT medium, frozen on dry ice, and stored at −80°C. OCT embedded tissue samples were cryosectioned (Microm HM550) into 40-μm-thick slices, collected as free-floating sections, and stored at −20°C in antifreeze medium until staining.

Postmortem tissues from the midbrain of patients with PD and controls were fixed in formalin, paraffin embedded, and stored at room temperature until sectioning. Paraffin-embedded samples were sectioned with a vibratome into 40-μm-thick sections and stored at 4°C until staining.

Human surgical resection samples were placed in a cryomold, covered with an OCT compound (Epredia Cryomatrix embedding resin, #67-690-06, Fisher Scientific), and snap frozen and stored at −80°C until cryosectioning. OCT embedded tissue samples were cryosectioned (Cryotome FE & FSE, A78910100, Thermo Fisher Scientific) into 20-μm slides and collected on Superfrost Plus Adhesion slides (#10149870, Thermo Fisher Scientific) and stored at −80°C until staining.

### Immunofluorescence

For IF on cell cultures derived from hiPSCs and from mESCs, cells were fixed with 4% PFA in 1× PBS to perform IF staining. We performed IF first to characterize each model and then to evaluate the changes after treatment using GFAP and S100β markers. In the 12-well plate, cells were plated on 18-mm coverslips coated with Matrigel for the monocultures or PLO and laminin for the mixed culture and then fixed with 4% PFA. To permeabilize the cells and reduce the nonspecific binding of the antibodies, cells were incubated with blocking solution with 10% donkey serum and 0.1% Triton for 1 hour. Later, cells were incubated with primary antibodies against the protein targets diluted (1:5) in blocking solution and stored at 4°C overnight. The following day, cells were washed three times with 1× PBS for 10 min each. Then, they were incubated with secondary antibodies conjugated with a fluorophore in blocking solution (1:5) in 1× PBS for 1 hour. After that, cells were washed three times with 1× PBS.

In the case of the IF on cell cultures derived from hNSCs, the incubations were performed in blocking buffer consisting of 10% normal goat serum (NGS) and 0.5% Triton X-100 in PBS (1 ml of NGS, 1 ml of 5% Triton X-100, and 8 ml of PBS). Cells were then processed as described above and incubated with specific primary and secondary antibodies listed in table S2.

In the case of the IF staining for TOMM20 and GFAP, it was performed by incubating cells with secondary antibodies anti-rabbit 488 and anti-chicken biotinylated in blocking solution (1:5). Later, cells were washed three times for 10 min each in 1× PBS and then incubated with Streptavidin 594 (1:200; Invitrogen, S11227) for 30 min followed by washing three times in 1× PBS for 10 min each. Last, they were incubated with DAPI (1:1000; Merck-Sigma, 32670) in 1× PBS for 5 min at room temperature and then washed three times for 10 min each with 1× PBS. The coverslips were then mounted on a glass slides sealed with Fluoromount-G (Invitrogen, 00-4958-02), letting them dry at room temperature in a dark box.

We adapted a published IF protocol for the staining experiments (*54*) for both cell cultures and postmortem human and tiger prefrontal cortex tissues. Postmortem human and tiger prefrontal cortex sections were immunostained free-floating.

For mouse brain tissue from the CCI experiment, free-floating sections were washed three times in 0.1 M PBS and then blocked at room temperature for 1 hour in PBS+ (10% donkey serum and 0.5% Triton X-100 in PBS). Primary antibody GFAP (Agilent, catalog no. Z0334, RRID:AB_10013382) was diluted in PBS+ and incubated with the brain sections overnight at 4°C. The next day, three washes with 0.1 M PBS were performed before and after incubation with DAPI and Alexa Fluor–conjugated secondary antibody diluted in PBS+ for 1 hour in room temperature. Brain sections were mounted on microscope slides, air dried, and coverslipped using FluorSave Reagent (Merck Millipore).

For IF on mouse brain tissue from the LPS injection experiment, brain sections were washed three times for 10 min in 0.02% Tris-buffered saline with Tween-20 (TBST, Surfact-Amps X-100, Thermo Fisher Scientific, ref. 28314) using 500 μl per well. Sections were then blocked in 5% NGS in 0.02% TBST for 1 hour at room temperature with gentle shaking. After blocking, sections were incubated overnight at 4°C with primary antibodies (1:500) diluted in 5% NGS: RFP-chicken (to enhance the TdTomato signal) (Rockland, ROCK600-901-379) and GFAP-mouse (Sigma-Aldrich, G3893), under gentle shaking. Following incubation, sections were washed three times for 10 min in TBST. Secondary antibody incubation was performed for 2 hours at room temperature (1:200) in 5% NGS with gentle shaking using Alexa Fluor 594 Goat anti-chicken (Invitrogen, ref. A32759) and Alexa Fluor 488 Goat anti-mouse (Jackson ImmunoResearch, 115-545-166). Thirty minutes before the end of incubation, DAPI (1:10,000; 25 μl of 1:500 stock in 500 μl) was added within the antibody mix. Sections were lastly washed three times with TBST and mounted in a solution of 80% glycerol and 20% water.

Immunohistochemistry for GFAP expression on BG were performed in postmortem human brains from age-paired patients with PD and non-PD patients. Brain sections were deparaffinized in xylene (5′) and a decreased concentration of ethanol from 100 to 50% (5′ each) and washed in PB. After antigen retrieval (121°C), endogenous peroxidases were blocked for 30′ in 3% H_2_O_2_ in TBS and unspecific peptides in 4% bovine serum albumin (BSA) and 0.02% Triton X-100 for 60 min. GFAP primary antibody (Dako, ref. Z0334) diluted 1:1000 was incubated overnight in blocking solution (4% BSA and 0.02% Triton X-100) at 4°C. After three washes in TBS with 0.01% Triton X-100, samples were incubated in biotinylated secondary antibody (mouse anti-rabbit 1:400) for 2 hours. After three washes in TBST, immunohistochemistry was developed in DAB (3,3′-diaminobenzidine tetrahydrochloride) substrate, following the manufacturer’s instruction (Thermo Fisher Scientific). Then, we performed dehydration in an increasing concentration of ethanol (from 50 to 100% plus xylene, each 5′).

IF on human surgical resection samples was performed as follows. Sections were fixed in 4% PFA in PBS for 15 min at room temperature, followed by permeabilization in 1% Triton-X in PBS for 15 min and blocking in blocking buffer (10% NGS and 2% BSA in PBS) for 2 hours. Sections were incubated with primary antibodies [anti-GFAP (#ab4674, Abcam), anti-Iba1 (#019-19741, FUJIFILM Wako Shibayagi), and anti-NeuN (#MAB377, Millipore)] in antibody diluent buffer (2% NGS in PBS) overnight at 4°C and visualized by secondary antibodies [Goat anti-Chicken IgY (H+L) Cross-Adsorbed Secondary Antibody Alexa Fluor Plus 594 (#A32759, Invitrogen), Goat anti-Mouse IgG (H+L) Cross-Adsorbed Secondary Antibody Alexa Fluor 488 (#A11001, Invitrogen), and Goat anti-Rabbit IgG (H+L) Highly Cross-Adsorbed Secondary Antibody Alexa Fluor Plus 647 (#A32733, Invitrogen)] in antibody diluent buffer for 2 hours at room temperature. Immunolabeled sections were counterstained with DAPI (1 μg/ml), washed, and mounted with Mowiol.

A comprehensive list of primary and secondary antibodies used in this study is in table S2.

### Image acquisition and processing

The images from the IF experiments on cell cultures from the hiPSCs and mESCs, postmortem human prefrontal cortex, postmortem tiger prefrontal cortex, and human surgical resection samples were acquired using a confocal microscope Nikon A1/R with different objectives (10×, 20×, and 40×, SISSA microscope facility) based on the analysis we performed. We acquired every image using three or four lasers with different wavelengths: FITC (488 nm), TRITC (564 nm), Cy5 (647 nm), and DAPI (408 nm). The images from the IF experiments on cell cultures from hNSCs were acquired using a spinning disk confocal microscope Nikon Eclipse Ti2-E inverted fully motorized microscope, equipped with CrestOptics CICERO spinning disk confocal module (50/250 μm) and Celesta 5-channel laser system. Images were processed with the Fiji/ImageJ software, merging the channels and producing a maximum *Z*-stack projection. Images from postmortem human PD samples (BG-CPP and BG-STN) were taken in an automated digital slide scanner Panoramic MIDI II by 3DHistech at the Achucarro’s Image Facility.

Images for quantification of the varicose projection astrocyte density after CCI in mice were acquired on a Nikon ECLIPSE Ti inverted microscope system using Volocity v7.0.0. Images were acquired using a 20× objective with focal planes 1.5 μm apart. For each animal, three sections containing visible lesion were selected. Three images were acquired around the lesion and equivalent areas were captured in the contralateral side. Representative images were acquired using a FLUOVIEW FV3000 confocal microscope. A 40× objective was used with focal planes 0.9 μm apart. Images from the medial side of the lesion, as well as equivalent contralateral and control regions, are shown.

Images for quantification of the varicose projection astrocyte density after LPS injections in mice were acquired on a Leica Stellaris 8 STED HT microscope. Images were acquired with a 20× objective; in selected cases, magnification was increased to 40× with 2× digital zoom to improve the resolution. Acquisition settings were as follows: resolution: 1024 × 1024; pinhole: 2; scan speed: 400; *Z*-step: 0.5 or 1.0 μm.

#### NF-κB translocation and astrocyte volume quantification

To quantify the NF-κB translocation and GFAP^+^ astrocyte volume, we treated astrocytes for 1 hour, and then we acquired three images for each coverslip (three coverslips for each treatment condition, *n* = 3). Images were processed using the “Volocity” software, considering the intensity in the cytoplasm and the nucleus of each astrocyte, and then we calculated the ratio of nuclei intensity over cytoplasm intensity to verify whether the translocation had occurred.

#### Varicose projection astrocyte quantification

To characterize the varicose projection astrocyte population, after treatment, we acquired large images for the 1-week-treated immature astrocytes (*n* = 3).

We selected three points for each coverslip in the respective three spatial coordinates centering a varicose projection. For each point, it was acquired at an area of 4 × 4 fields with a 20× magnification; this resulted in three large images for each coverslip, nine images for each condition at 1 week (Ctr, IL-1β, TNF-α, and IL-1β–TNF-α) with 20 μm of *Z*-stack with 19 steps, each 1 μm. Images were processed into the Fiji/ImageJ software, merging the channels, producing a maximum *Z*-stack projection, and exporting them as a TIFF file for the count. Varicosities were counted using the “Cell Counter” plug-in in Fiji/ImageJ. We identified varicose projections along their corresponding originating cells.

Varicose projection astrocytes were operationally defined as astrocytes exhibiting two or more regularly spaced, bead-like varicosities along one or more discernible astrocytic processes, identified by GFAP or S100β positivity. The process length was not used as a strict exclusion criterion as astrocytic morphology differs substantially between in vivo and in vitro contexts; instead, the defining feature used for classification was the presence of varicosities along astrocytic processes. Varicose projection astrocytes were only scored when elongated processes could be unambiguously traced to a single astrocyte soma. Structures that could not be confidently assigned were excluded from analysis. Astrocytes displaying uniformly thickened, irregular, or diffusely “bumpy” processes without discrete varicosities were not classified as varicose projection astrocytes. When necessary, the total number of cells in each large image was calculated using the “Volocity” software with DAPI staining. Subsequently, we measured the percentage of varicose projection astrocytes either over the total number of nuclei in the large images or over the total number of GFAP^+^ astrocytes. Whether varicose projection astrocyte density was measured over the total nuclei or total number of GFAP^+^ astrocytes was always specified per each specific case throughout the Results section.

### Statistical analysis

For NF-κB translocation and varicose projection astrocyte counts, data were obtained from at least three independent biological replicates per type of experiment. For in vitro experiments, biological replicates corresponded to independent differentiation and treatment experiments performed on separate cultures; for in vivo analyses, biological replicates corresponded to independent animals or human samples, as specified in the figure legends. Before statistical analysis, data were tested for normality using the Shapiro-Wilk test. The values were then organized and exported into the GraphPad Prism 8 software for statistical analysis. Depending on the comparison, results were analyzed using one-way analysis of variance (ANOVA) followed by Tukey’s multiple comparisons test or a two-tailed Student’s *t* test. Statistical significance was set at *P* < 0.05 (*P < 0.05; **P ≤ 0.01; ***P < 0.001), and data are presented as means ± SEM.

## Supplementary Materials

### The PDF file includes:

Tables S1 and S2 Figs. S1 to S12 Legend for supplementary raw data

### Other Supplementary Material for this manuscript includes the following:

Supplementary Raw Data
