# Supplementary material for: Varicose projection astrocytes: Conserved reactive cells in brain pathology
Source: Sci Adv. 2026 Jul 17;12(29):eady8204. doi: 10.1126/sciadv.ady8204 (PMC13378549; doi:10.1126/sciadv.ady8204)
Supplement: Supplementary file 1 — Tables S1 and S2 Figs. S1 to S12 Legend for supplementary raw data [file sciadv.ady8204_sm.pdf]

Science Advances
AAAS

# Supplementary Materials for

## Varicose projection astrocytes: Conserved reactive cells in brain pathology

Caterina Ciani *et al.*

Corresponding author: Carmen Falcone, cfalcone@towson.edu

*Sci. Adv.* **12**, eady8204 (2026)
DOI: 10.1126/sciadv.ady8204

**The PDF file includes:**

Tables S1 and S2
Figs. S1 to S12
Legend for supplementary raw data

**Other Supplementary Material for this manuscript includes the following:**

Supplementary Raw Data

**Table S1. List of human samples used in this study.**

| ID | Sex | Age (y) | Brain region | Type of tissue | Pathology |
|---|---|---|---|---|---|
| 1 | M | 77 | PFC | Postmortem | Control |
| 2 | F | 81 | PFC | Postmortem | Control |
| 3 | M | 77 | PFC | Postmortem | Control |
| 4 | F | 79 | PFC | Postmortem | AD |
| 5 | F | 84 | PFC | Postmortem | AD |
| 6 | F | 88 | PFC | Postmortem | AD |
| 7 | F | 79 | PFC | Postmortem | PD |
| 8 | M | 83 | PFC | Postmortem | PD |
| 9 | F | 89 | PFC | Postmortem | PD |
| 10 | M | 78 | PFC | Postmortem | MS |
| 11 | F | 81 | PFC | Postmortem | MS |
| 12 | M | 78 | PFC | Postmortem | MS |
| 13 | F | 68 | STN | Postmortem | Control |
| 14 | M | 64 | STN | Postmortem | Control |
| 15 | F | 56 | STN | Postmortem | Control |
| 16 | M | 81 | STN | Postmortem | PD |
| 17 | F | 70 | STN | Postmortem | PD |
| 18 | M | 68 | STN | Postmortem | PD |
| 19 | F | 68 | BG | Postmortem | Control |
| 20 | M | 64 | BG | Postmortem | Control |
| 21 | F | 56 | BG | Postmortem | Control |
| 22 | M | 81 | BG | Postmortem | PD |
| 23 | F | 70 | BG | Postmortem | PD |
| 24 | M | 68 | BG | Postmortem | PD |

| | | | | | |
|---|---|---|---|---|---|
| 25 | M | 60 | Cortex | Surgical resection | Control |
| 26 | M | 61 | Cortex | Surgical resection | Control |
| 27 | M | 31 | Cortex | Surgical resection | Control |
| 28 | F | 28 | Cortex | Surgical resection | Ep/Hsc |
| 29 | F | 27 | Cortex | Surgical resection | Ep/Hsc |
| 30 | M | 47 | Cortex | Surgical resection | Ep/Hsc |
| 31 | F | 37 | Cortex | Surgical resection | Ep/T |
| 32 | M | 55 | Cortex | Surgical resection | Ep/T |
| 33 | M | 20 | Cortex | Surgical resection | Ep/T |

Abbreviations: M=male; F=female; y=years; PFC=Prefrontal cortex; STN: Subthalamic nucleus; BG= Basal ganglia; AD= Alzheimer's disease; PD= Parkinson' disease; MS=Multiple sclerosis; Ep/Hsc= Focal structural epilepsy with hippocampus sclerosis; Ep/T=Cortical epilepsy due to CNS tumour.

**Table S2. Antibody list.**

| Antibody | Species | Dilution | Company, Catalog # |
|---|---|---|---|
| GFAP | Rabbit | 1:500 | Agilent, Z033401-2 |
| GFAP | Mouse | 1:500 | Abcam, AB190288 |
| GFAP | Mouse | 1:500 | Sigma Aldrich, G3893 |
| GFAP | Chicken | 1:500 | Invitrogen, PA1-10004 |
| GFAP | Chicken | 1:1000 | Sigma Aldrich, AB5541 |
| S100β | Rabbit | 1:300/1:500 | Abcam, AB52642 |
| S100β | Mouse | 1:300/1:500 | Sigma Aldrich, S2532 |
| ALDH1L1 | Rabbit | 1:500 | Abcam, AB190298 |
| ALDH1L1 | Mouse | 1:250 | Antibodiesinc, 75-164-020 |
| AQP4 | Rabbit | 1:400 | Sigma Aldrich, AB3594 |
| SOX9 | Rabbit | 1:500 | Thermo Fisher, 7H13L8 |
| SOX9 | Rabbit | 1:1000 | Sigma Aldrich, AB5535-25UG |
| Kir4.1 | Rabbit | 1:500 | Abcam, AB240876 |
| Vimentin | Mouse | 1:200 | Abcam, AMAB8069 |
| GLAST/EAAT1 | Rabbit | 1:500 | Novus Bio, NB100-1869 |
| GLAST/EAAT1 | Rabbit | 1:1000 | Synaptic Systems, 250113 |
| GS | Rabbit | 1:500 | Abcam, AB73593 |
| MAP2 | Rabbit | 1:500 | Cell Signaling, 8707S\D5G1 |
| NF-κB p65 | Rabbit | 1:1000 | Cell signaling, D14E12/ 8242S |
| TOMM20 rabbit | Rabbit | 1:1000 | Abcam, AB186735 |
| CD49f | Rat | 1:1000 | Thermo Fisher, 14-0495-82 |
| CD63 | Mouse | 1:250 | Thermo Fisher, 10628D |
| CD9 | Mouse | 1:100 | Thermo Fisher, 10626D |
| Integrin β1 | Rabbit | 1:100 | CST, 349715 |
| Calnexin | Rabbit | 1:500 | Abcam, AB10286 |
| M6PR | Mouse | 1:500 | Abcam, AB2733 |

| | | | |
|---|---|---|---|
| RFP | Chicken | 1:500 | Rockland, ROCK600-901-379 |
| anti-rabbit Alexa Fluor 488 | Donkey | 1:400 | Invitrogen, 21206 |
| anti-mouse Alexa Fluor 594 | Donkey | 1:400 | Invitrogen, 21203 |
| anti-mouse Alexa Fluor 488 | Donkey | 1:400 | Invitrogen, 21202 |
| anti-rabbit Alexa Fluor 594 | Donkey | 1:400 | Invitrogen, 21209 |
| anti-chicken Biotinylated | Donkey | 1:200 | Invitrogen, SA1-72003 |
| Anti-chicken | Goat | 1:200 | Invitrogen, A32759 |
| Anti-mouse | Goat | 1:200 | Jackson ImmunoResearch, 115-545-166 |

**Supplementary figures**

**Fig. S1.**

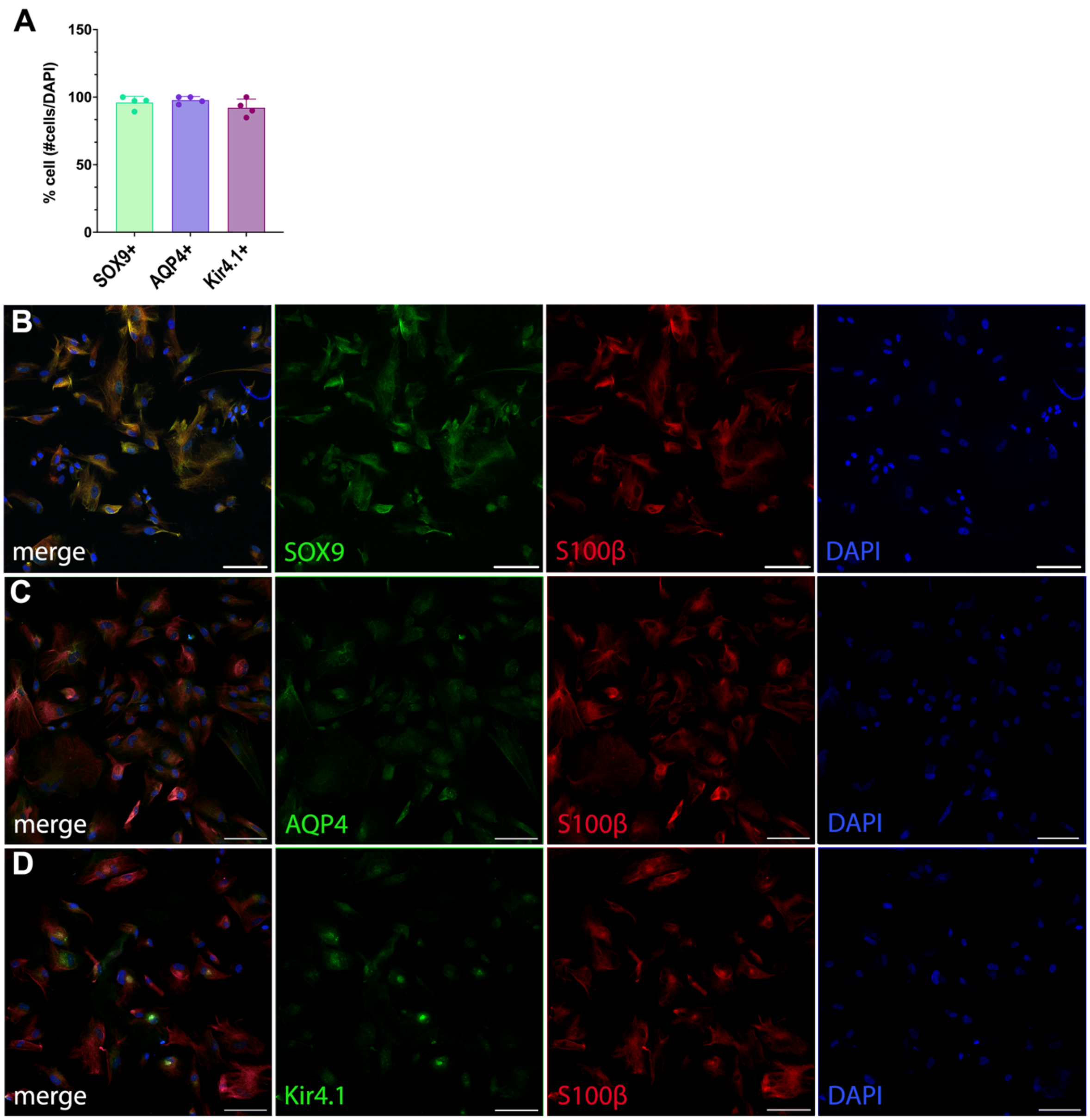


**Fig. S1. Characterization of hiPSC-derived astrocytes. (A)** Quantification of SOX9+, AQP4+, and Kir4.1+ cells calculated as number of positive cells over DAPI. (**B–D**) Representative immunofluorescence images showing the expression of key astrocytic markers in human induced pluripotent stem cell (hiPSC)-derived immature astrocytes (n = 3). (**B**) SOX9 (green) (**C**) AQP4 (green), and (**D**) Kir4.1 (green) are co-stained with the astrocytic marker S100β (red) and nuclear

marker DAPI (blue). Individual channels highlighting the expression pattern of SOX9, AQP4, and Kir4.1 in astrocytes. SOX9 is predominantly localized in the nuclei and perinuclear region, AQP4 exhibits nuclear expression with lower levels in the astrocytic membrane, and Kir4.1 is enriched in the nuclear region. S100β (red) effectively delineates the morphology of hiPSC-derived astrocytes. DAPI (blue) stains cell nuclei. Scale bars = 100 μm. These findings confirm the astrocytic identity of hiPSC-derived astrocytes based on the expression of multiple astrocytic markers.

**Fig. S2.**

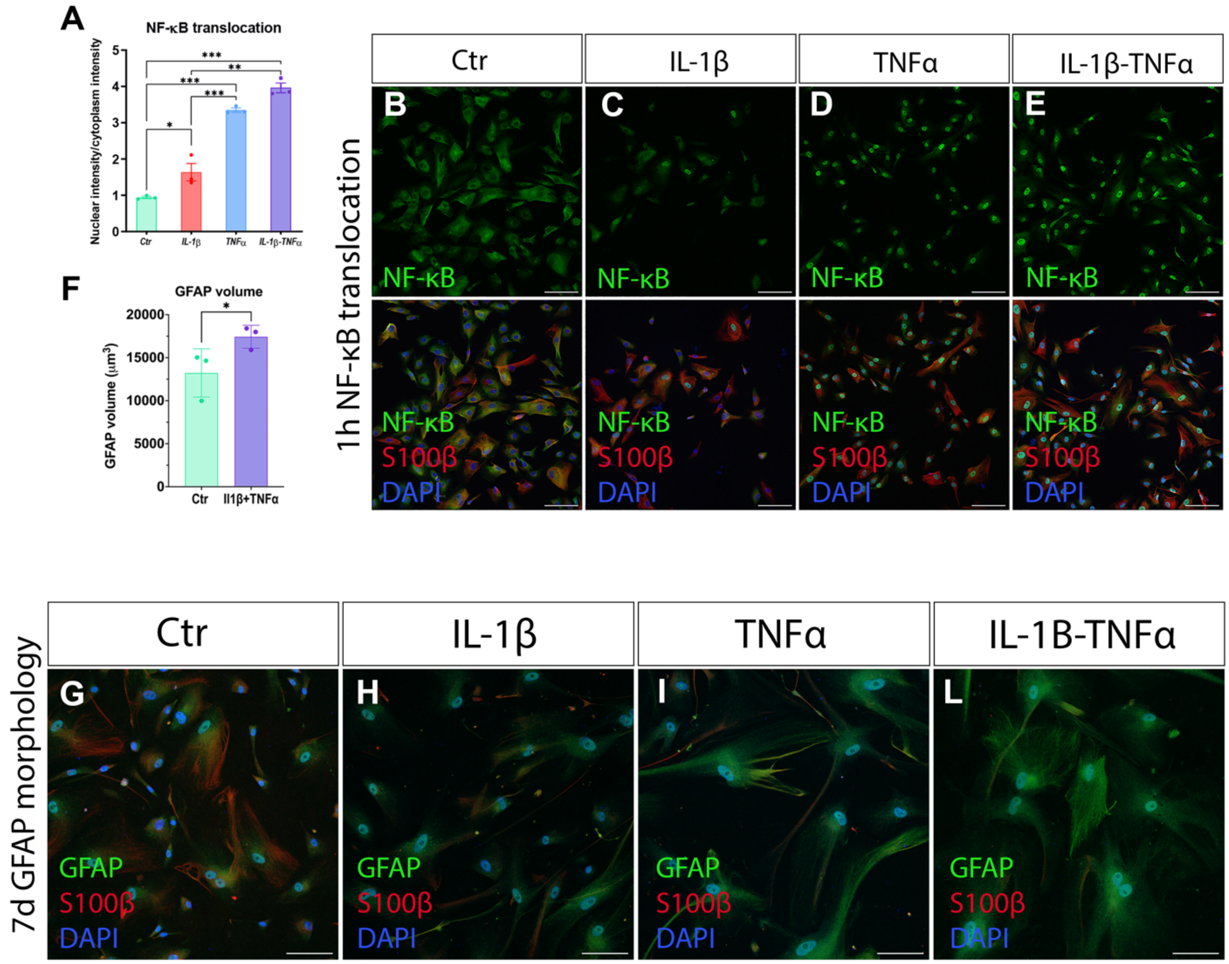


**Fig. S2. Cytokine treatments and reactivity of hiPSC-derived astrocytes.** (**A**) Quantification of NF-κB nuclear translocation, assessed by the nuclear-to-cytoplasmic intensity ratio in each condition ($n = 3$; $p < 0.05$, *$p \leq 0.001$, **$p < 0.0001$; one-way ANOVA with Tukey's multiple comparisons test). (**B–E**) Representative immunofluorescence images showing NF-κB (green) localization under different cytokine treatment conditions. (**B**) In control conditions, NF-κB remains entirely cytoplasmic. (**C**) In IL-1β-treated astrocytes, NF-κB is partially localized in both the cytoplasm and nucleus. (**D**) In TNF-α-treated astrocytes, NF-κB is predominantly nuclear. (**E**) In the combined IL-1β + TNF-α condition, NF-κB is almost entirely nuclear. Merged images show NF-κB (green), S100β (red), and DAPI (blue) across conditions. (**F**) Quantification of GFAP

volume in $um^3$ in control versus IL-1β + TNF-α-treatment condition ($n = 3$; $p < 0.05$, *$p \leq 0.001$, **$p < 0.0001$; one-way ANOVA with Tukey's multiple comparisons test). (**G**) Control condition, showing resting astrocyte morphology. This panel is re-used in" Fig. 2C-panel Ctr-7d" showing the same condition. (**H**) IL-1β-treated astrocytes exhibit a slightly enlarged soma. (**I**) TNF-α-treated astrocytes display more pronounced soma enlargement and elongated processes. (**J**) IL-1β + TNF-α-treated astrocytes exhibit the most evident morphological changes, including pronounced cell enlargement and extended processes. Scale bars = 100 μm. These findings confirm that NF-κB nuclear translocation and astrocyte morphological changes are hallmarks of inflammatory reactivity in hiPSC-derived astrocytes.

**Fig. S3.**

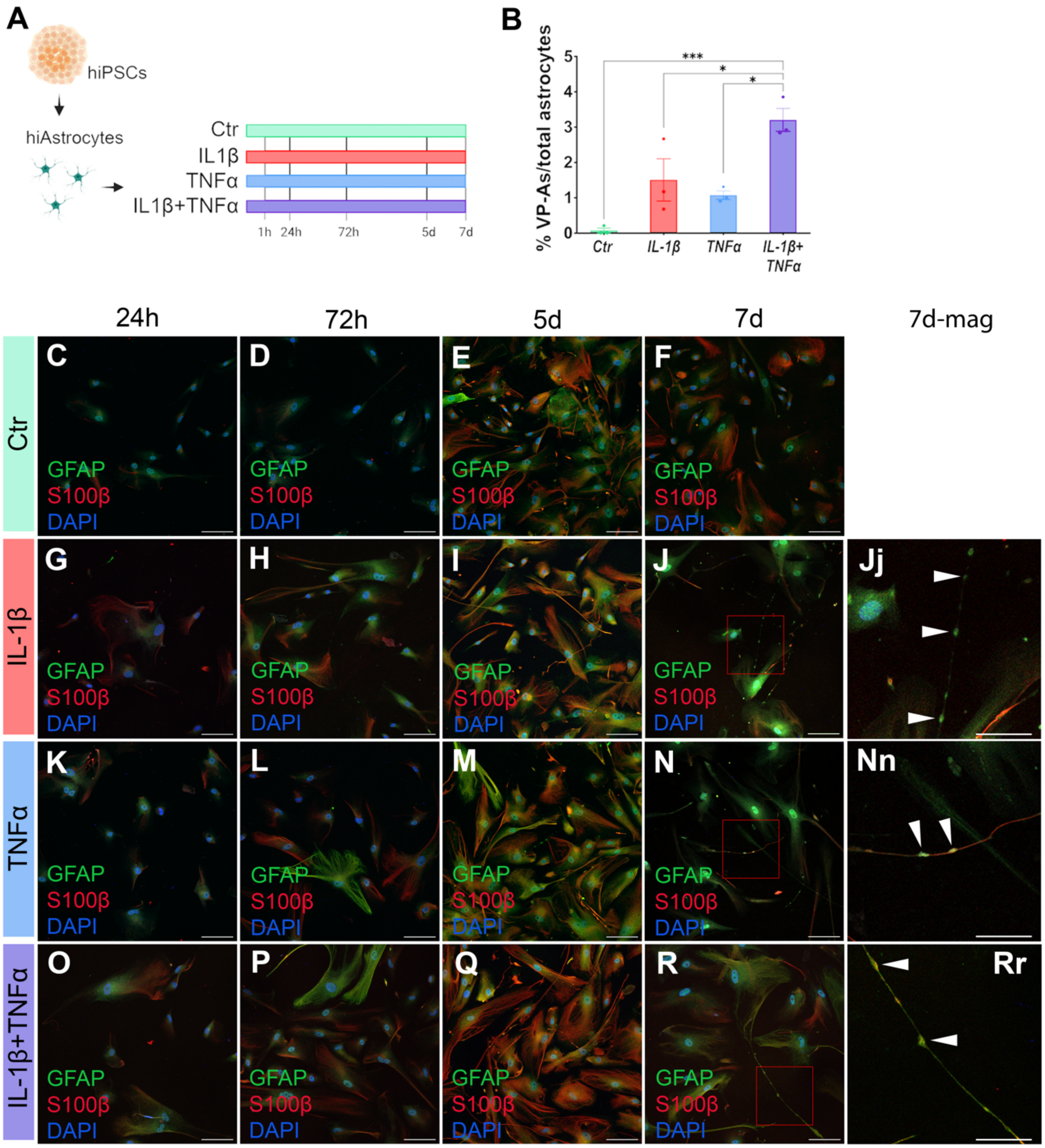


**Fig. S3. Varicose projection astrocytes are induced by pro-inflammatory cytokines in human astrocytes.** (**A**) Schematic representation of experimental protocol. (**B**) Quantification of varicose projection astrocyte density as a percentage of total astrocytes in culture across conditions. Data are mean ± s.e.m., statistical significance assessed by one-way ANOVA with Tukey's multiple comparisons test ($n = 3$; * $p < 0.05$, ** $p < 0.01$, *** $p < 0.001$). (**C–F**) Representative

immunofluorescence images of astrocytes under control (Ctr) conditions at (C) 24 hours, (D) 72 h, (E) 5 days, and (F) 7 d, showing the absence of varicose projection astrocytes (n = 3). h= hours; d= days. (**G–Jj**) Representative images of astrocytes treated with IL-1β at corresponding time points, demonstrating the progressive emergence of varicose projection astrocytes, particularly at 5 d and 7 d (n=3). (**Jj**) Higher magnification of the boxed region in (**J**), highlighting varicose projection astrocytes. (**K-Nn, O-Rr**) Representative images of astrocytes treated with TNF-α (n=3, **K-Nn**) and IL-1β + TNF-α (n=3, **O-Rr**) at corresponding time points, showing an increased frequency of varicose projection astrocytes, most notably in the combined condition (**Nn**) Higher magnification of the boxed region in (**N**), highlighting varicose projection astrocytes. (**Rr**) Higher magnification of the boxed region in (**R**), highlighting varicose projection astrocytes. In all the fluorescent images, GFAP, S100β, and DAPI staining in green, red, and blue, respectively. White arrowheads point to varicosities. Scale bars (C-F, G-J, K-N, O-R) = 100 μm. Scale bars (Jj,Nn,Rr) = 50 μm.

**Fig. S4.**

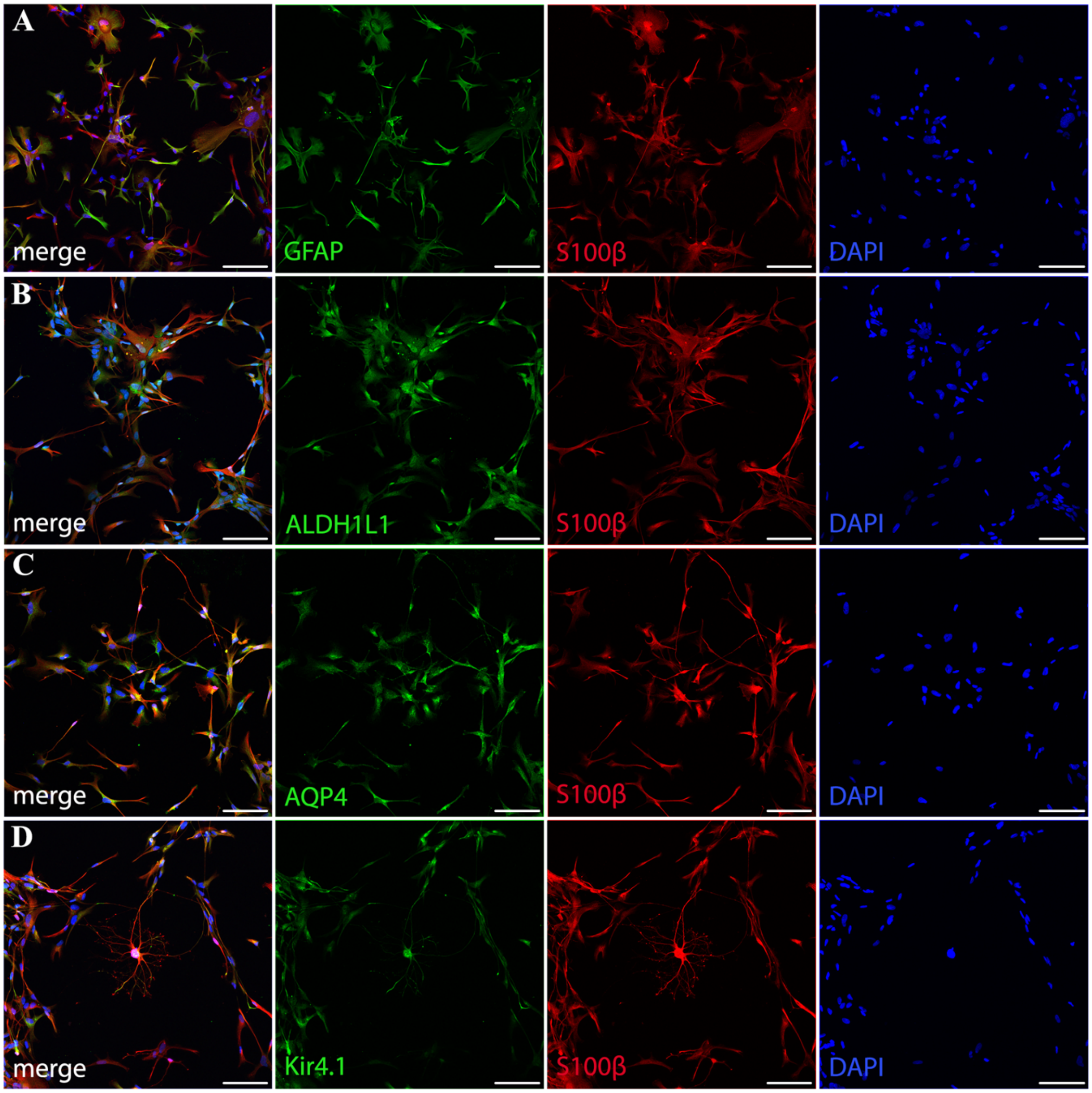


**Fig. S4. Characterization of hiPSC-derived astrocytes in mixed culture (1).** Representative immunofluorescence images showing the expression of astrocytic markers in human induced pluripotent stem cell (hiPSC)-derived astrocytes within mixed cultures (n = 3). (**A**) GFAP (green), (**B**) ALDH1L1 (green), (**c**) AQP4 (green), and (**D**) Kir4.1 (green) are co-stained with the astrocytic marker S100β (red) and nuclear marker DAPI (blue). Individual channels highlighting the expression pattern of GFAP, ALDH1L1, AQP4, and Kir4.1. GFAP is more pronouncedly

expressed in the cytoplasm, ALDH1L1 is present in the astrocytic cytoplasm, AQP4 shows increased expression in both the cytoplasm and membrane, and Kir4.1 is distinctly localized in the astrocytic cytoplasm and membrane. S100β (red) effectively delineates astrocyte morphology, appearing more defined compared to hiPSC-derived immature astrocytes. DAPI (blue) stains cell nuclei. Scale bars = 100 μm. These findings confirm the astrocytic identity of hiPSC-derived astrocytes in mixed cultures.

**Fig. S5.**

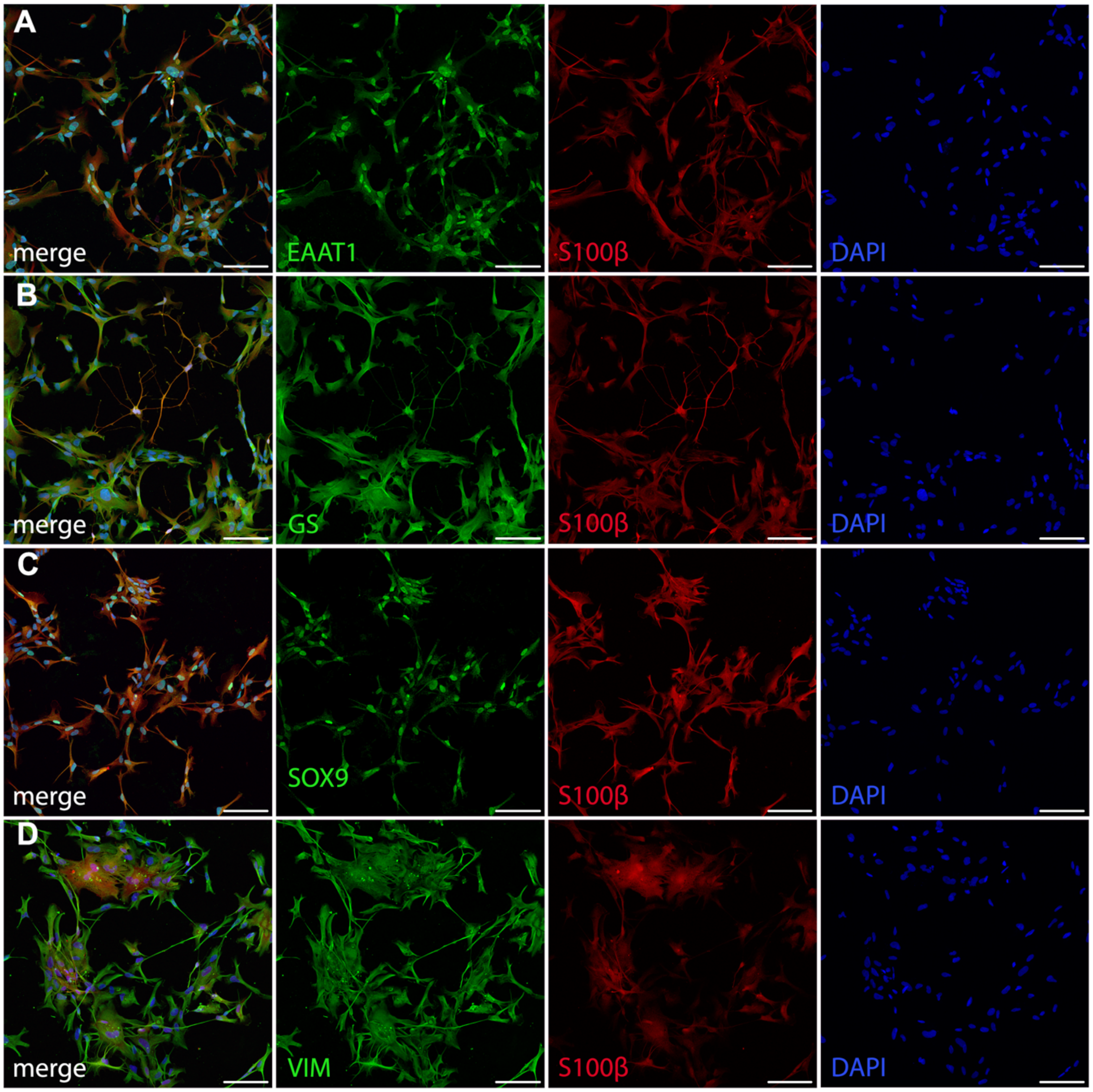


**Fig. S5. Characterization of hiPSC-derived astrocytes in mixed culture (2).** Representative immunofluorescence images showing the expression of astrocytic markers in human induced pluripotent stem cell (hiPSC)-derived astrocytes within mixed cultures (n = 3). (**A**) EAAT1 (green), (**B**) GS (green), (**C**) SOX9 (green), and (**D**) VIM (green) are co-stained with the astrocytic marker S100β (red) and nuclear marker DAPI (blue). Individual channels highlighting the expression pattern of EAAT1, GS, SOX9, and VIM. EAAT1 is localized in both nuclei and cell

processes, GS is strongly expressed in the cytoplasm, SOX9 is detected in both cytoplasmic and nuclear compartments with more pronounced nuclear staining, and VIM outlines the morphology of hiPSC-derived astrocytes. S100β (red) effectively delineates astrocyte morphology. DAPI (blue) stains cell nuclei. Scale bars = 100 μm. These findings confirm the astrocytic identity of hiPSC-derived astrocytes in mixed cultures.

**Fig. S6.**

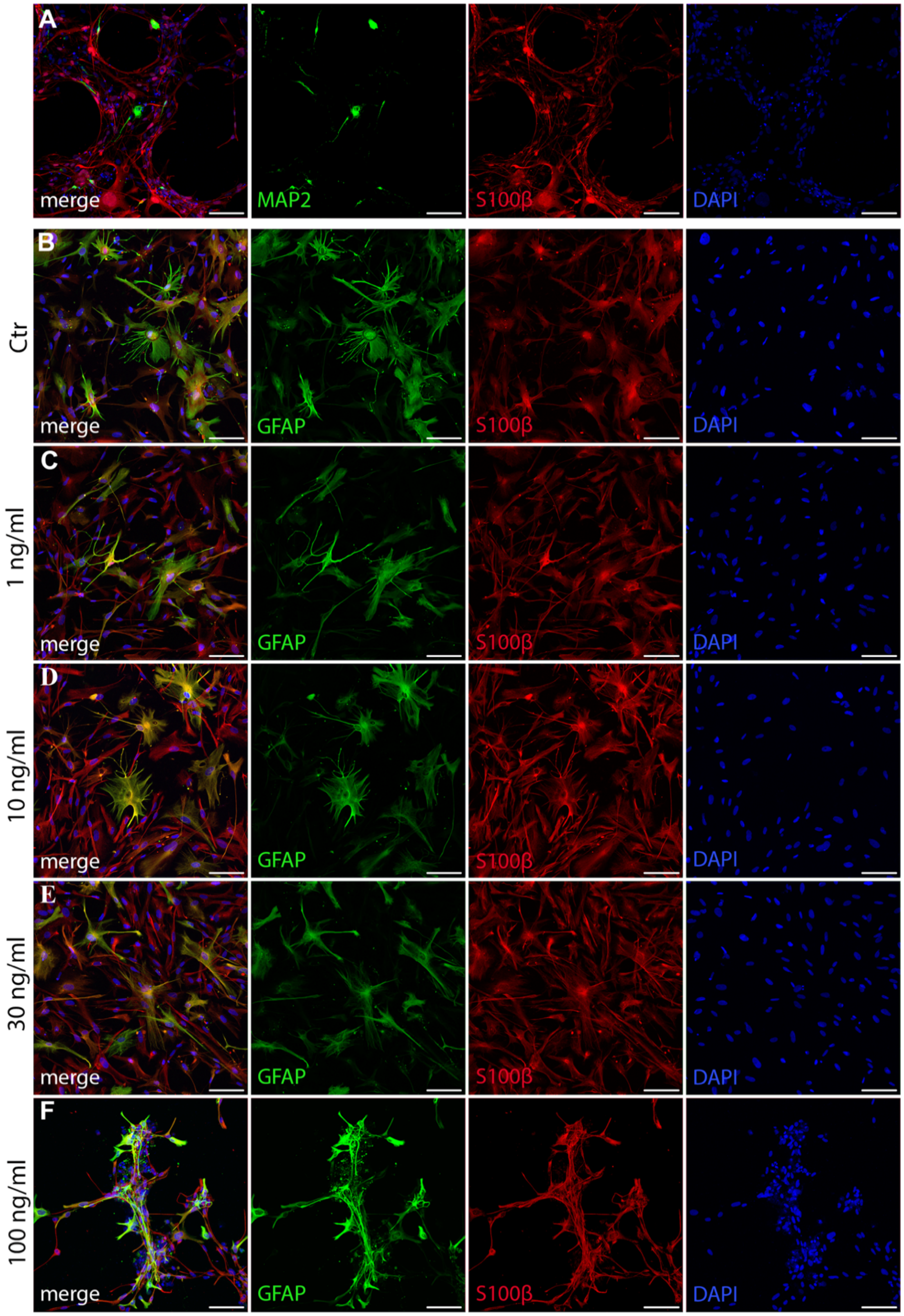
A
merge
MAP2
S100β
DAPI
B
Ctr
GFAP
C
1 ng/ml
D
10 ng/ml
E
30 ng/ml
F
100 ng/ml

**Fig. S6. Cytokine treatments and reactivity of hiPSC-derived astrocytes in mixed culture.** (**A**) Representative immunofluorescence image showing the presence of neurons in mixed cultures, confirmed by staining for MAP2 (green), S100β (red), and DAPI (blue). Individual channels highlighting MAP2, S100β, and DAPI. (**B–F**) Representative immunofluorescence images showing astrocyte morphology under different cytokine treatment conditions. (**B**) Control condition, with merged staining for GFAP (green), S100β (red), and DAPI (blue). Individual channels show GFAP, S100β, and DAPI in the control condition. (**C–F**) Cytokine treatments with increasing concentrations of IL-1β + TNF-α (1 ng/mL to 100 ng/mL), showing progressive astrocyte reactivity. (**C**) 1 ng/mL treatment, (**D**) 10 ng/mL treatment, (**E**) 30 ng/mL treatment, (**F**) 100 ng/mL treatment. GFAP staining highlights astrocyte morphology changes, S100β marks astrocytes, and DAPI stains nuclei. Scale bars = 100 µm. n = 3. These findings confirm astrocyte reactivity and morphological changes in response to increasing cytokine concentrations in mixed cultures.

**Fig. S7.**

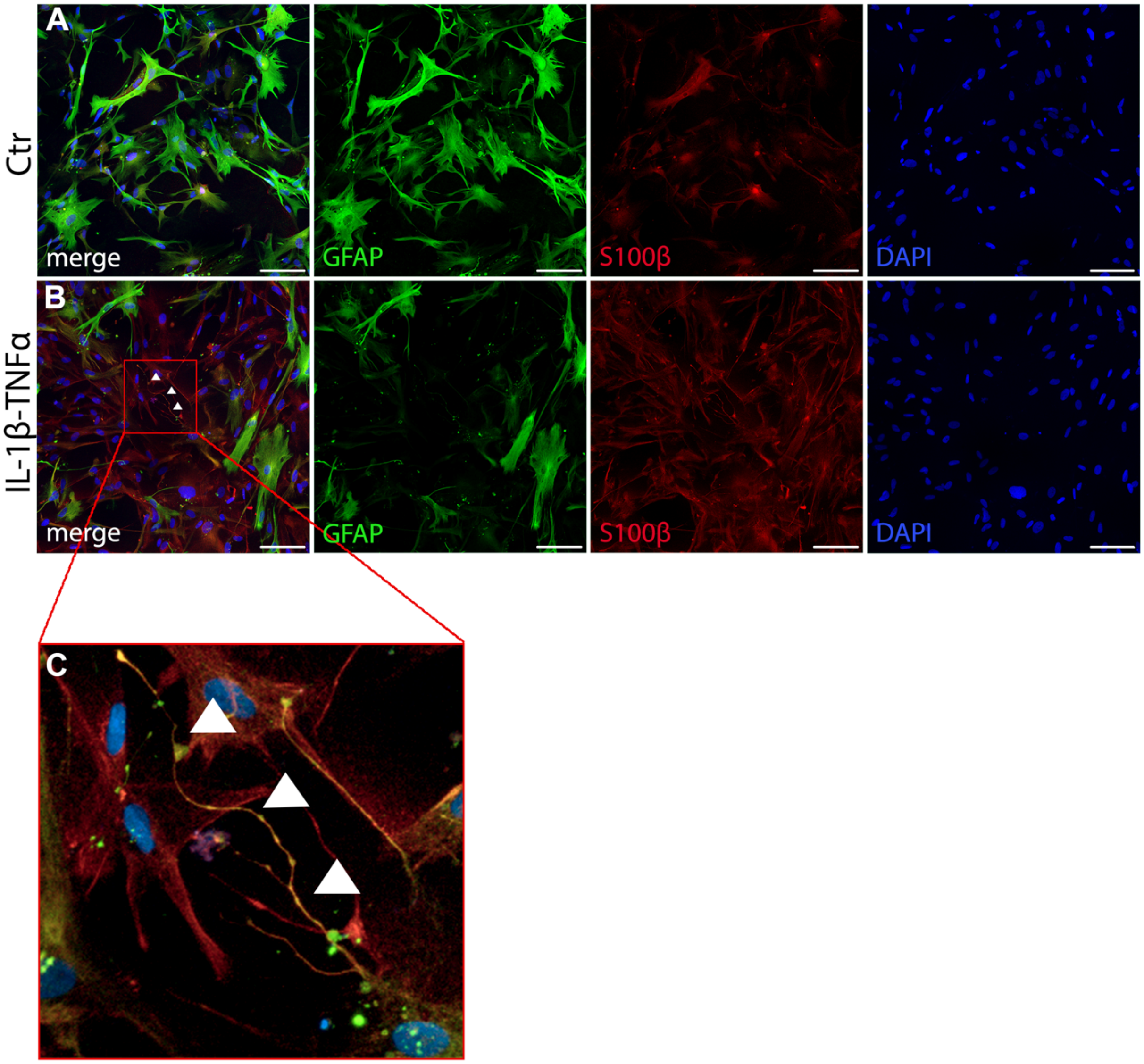


**Fig. S7. Varicose-projection astrocytes in treated human astrocytes in mixed culture.** (**A**) Representative immunofluorescence image of astrocytes in the control condition, with merged staining for GFAP (green), S100β (red), and DAPI (blue). Individual channels highlight GFAP, S100β, and DAPI in control astrocytes. (**B**) Astrocytes treated with IL-1β (10 ng/mL) + TNF-α (10 ng/mL) for 7 days, showing the presence of varicose projections (arrowheads). Merged staining for GFAP (green), S100β (red), and DAPI (blue). Individual channels highlight GFAP, S100β, and DAPI in treated astrocytes, with white arrowheads indicating varicose projections. (**C**) Higher magnification of a varicose projection, with arrowheads marking its structure. n = 3. Scale

bars = 100 μm. These findings confirm the presence of varicose-projection astrocytes in cytokine-treated human astrocytes within mixed cultures.

**Fig. S8.**

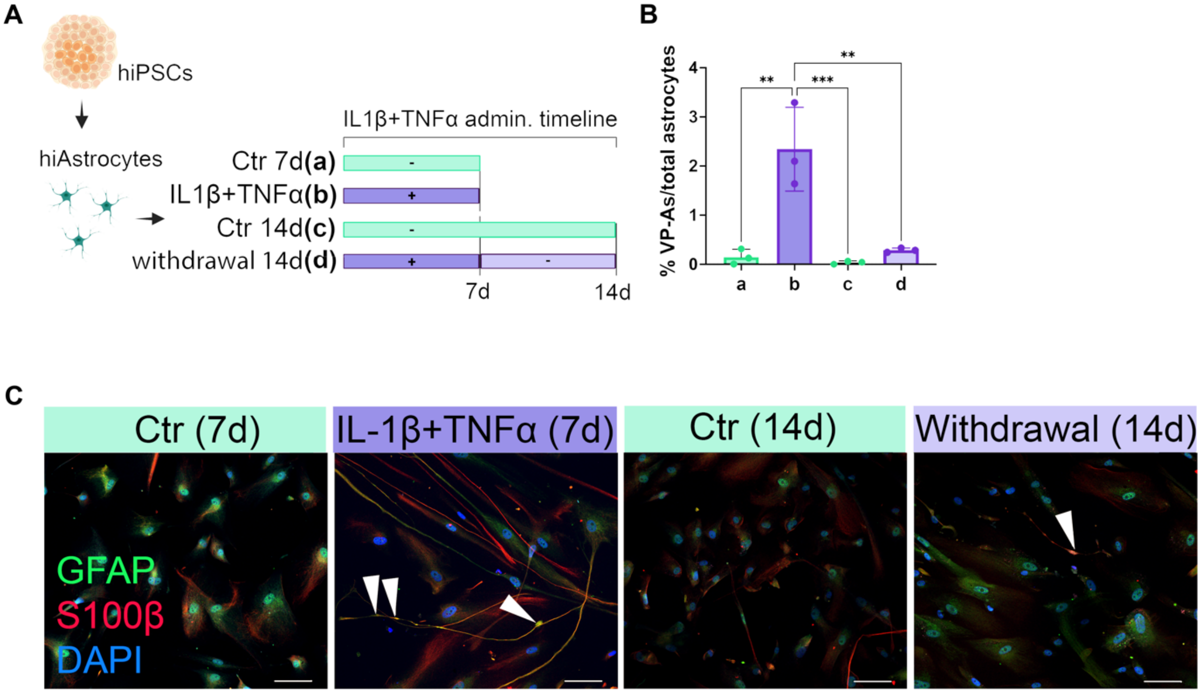


**Fig. S8. Varicose projection astrocytes reverse after removing the cytokine exposure.** (**A**) Schematic of the protocol. Human iPSC-derived astrocytes were treated with IL-1β and TNF-α for 7 days, followed by cytokine withdrawal for other 7 days to assess reversibility. Four conditions were analyzed: (a) control at 7 days, (b) IL-1β + TNF-α treatment at 7 days, (c) control at 14 days, and (d) IL-1β + TNF-α withdrawal (rescue) at 14 days. (**B**) Quantification of varicose projection astrocytes as a percentage of total astrocytes in each condition. Data are mean ± s.e.m., statistical significance assessed by one-way ANOVA with Tukey's multiple comparisons test ($n = 3$, * $p < 0.05$, ** $p < 0.01$, *** $p < 0.001$). (**C**) Representative immunofluorescence images of astrocytes [GFAP (green), S100β (red), and DAPI (blue)]. Varicose projection astrocytes were identified based on the presence of varicosities along astrocytic processes, independent of absolute process length. Scale bars (C) = 50 µm. White arrowheads point to varicosities.

**Fig. S9.**

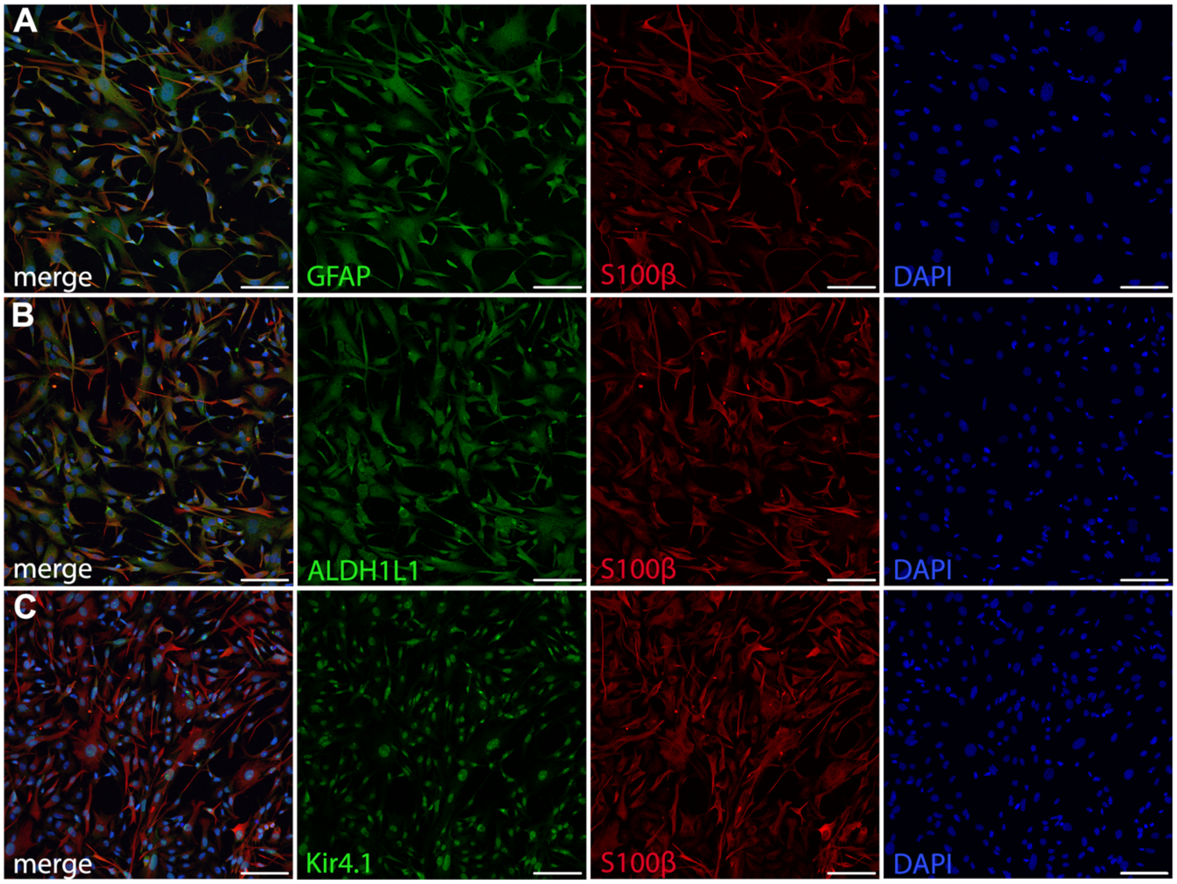


**Fig. S9. Characterization of ESCs-derived mouse astrocytes 1.** Representative immunofluorescence images showing the expression of key astrocytic markers in embryonic stem cell (ESC)-derived mouse astrocytes (n = 3). (**A**) GFAP (green), (**B**) ALDH1L1 (green), and (**C**) Kir4.1 (green) are co-stained with the astrocytic marker S100β (red) and nuclear marker DAPI (blue). Individual channels highlight the expression pattern of GFAP, ALDH1L1, and Kir4.1. GFAP is localized in the cytoplasm, ALDH1L1 is expressed in the astrocytic cytoplasm, and Kir4.1 is predominantly found in the nuclear region. S100β (red) effectively delineates the morphology of ESC-derived astrocytes. DAPI (blue) stains cell nuclei. Scale bars = 100 μm. These findings confirm the astrocytic identity of ESC-derived mouse astrocytes based on the expression of multiple astrocytic markers.

**Fig. S10.**

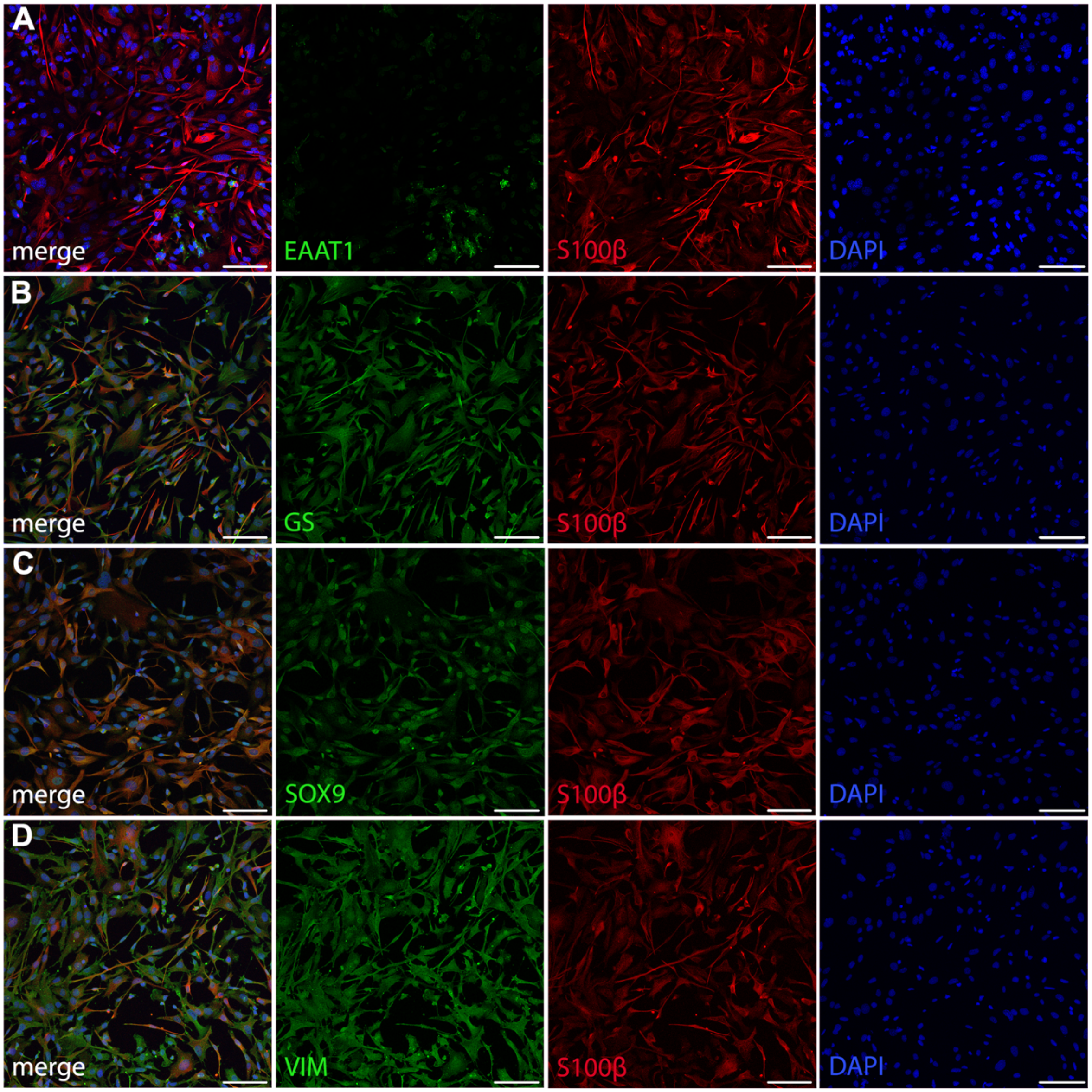


**Fig. S10. Characterization of ESCs-derived mouse astrocytes 2.** Representative immunofluorescence images showing the expression of additional astrocytic markers in embryonic stem cell (ESC)-derived mouse astrocytes (n = 3). (**A**) EAAT1 (green), (**B**) GS (green), (**C**) SOX9 (green), and (**D**) VIM (green) are co-stained with the astrocytic marker S100β (red) and nuclear marker DAPI (blue). Individual channels highlight the expression pattern of EAAT1, GS, SOX9, and VIM. EAAT1is predominantly localized in the nucleus with faint expression in cell processes, GS is strongly expressed in the cytoplasm, SOX9 is detected in both the nucleus and cytoplasm,

and VIM delineates the morphology of ESC-derived astrocytes. S100β (red) effectively marks the morphological features of ESC-derived astrocytes. DAPI (blue) stains cell nuclei. Scale bars = 100 μm. These findings further confirm the astrocytic identity of ESC-derived mouse astrocytes based on the expression of multiple astrocytic markers.

**Fig. S11.**

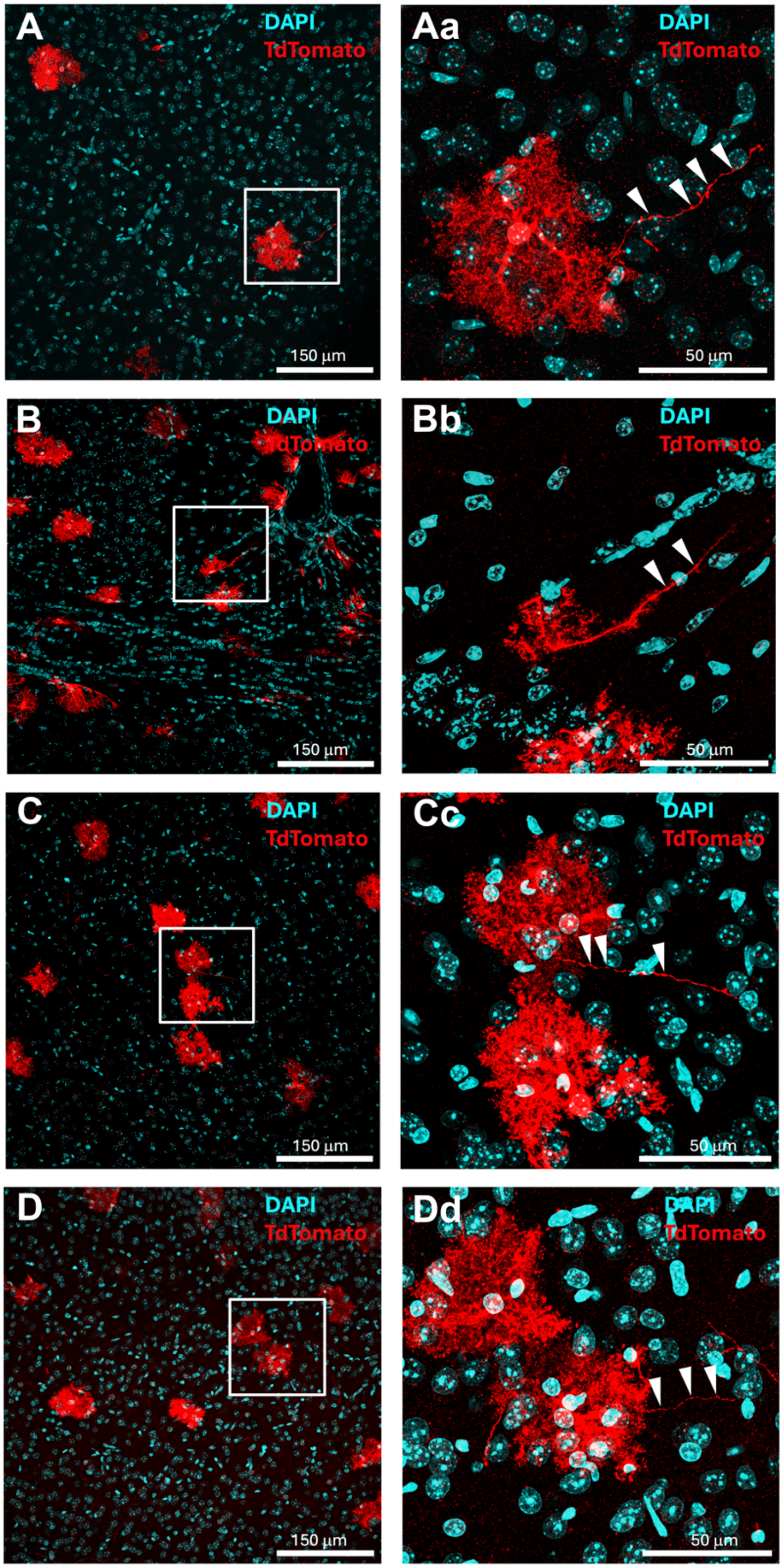
A
DAPI
TdTomato
150 μm
Aa
DAPI
TdTomato
50 μm
B
DAPI
TdTomato
150 μm
Bb
DAPI
TdTomato
50 μm
C
DAPI
TdTomato
150 μm
Cc
DAPI
TdTomato
50 μm
D
DAPI
TdTomato
150 μm
Dd
DAPI
TdTomato
50 μm

**Fig. S11. Varicose-projection astrocytes in a mouse model of inflammation.** Additional representative images of sparsely TdTomato-labeled varicose projection astrocytes (red) from LPS-treated mice from the experiment shown in Fig. 5K-O (n=8) . DAPI is in cyan. (**A,B,C,D**) images were taken at a 20X objective on a confocal microscope. (**Aa,Bb,Cc,Dd**) zoomed-in images of white squares in A,B,C and D respectively, taken at a 40X objective. Scale bars (A,B,C,D) = 150 µm. Scale bars (Aa,Bb,Cc,Dd) = 50 µm. Arrowheads point to varicosities.

**Fig. S12**

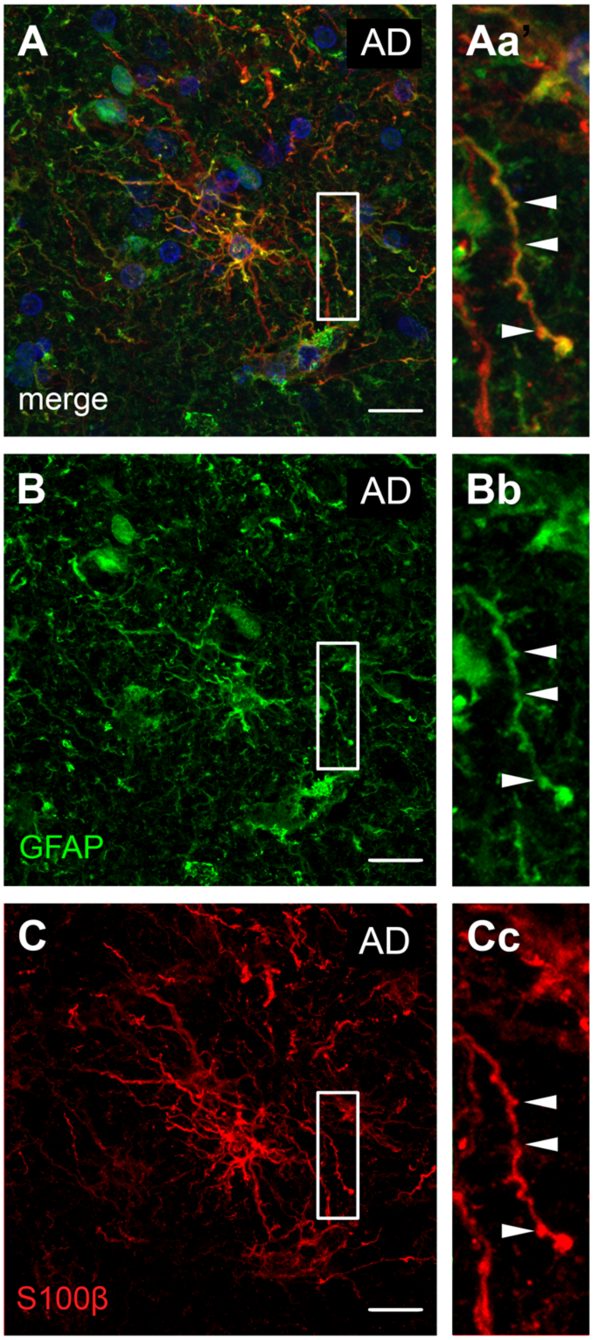


**Fig. S12. Varicose projection astrocytes express both GFAP and S100β.** Representative immunofluorescence images showing varicose projection astrocytes stained with GFAP (green),

S100β (red) and DAPI (blue) (n = 3). (**A**) merge, (**B**) GFAP only, (**C**) S100β only. (**Aa, Bb, C**c) Higher magnification of the boxed regions in (A), (B), and (C), respectively. Arrowheads point to varicosities. Scale bars = 30 μm.

**Supplementary Raw Data**

This spreadsheet contains the raw data referenced in the figures. Data are organised one dataset per sheet, with each sheet named and corresponding to the following figure panels: Fig. 2C, 2F, 3B, 5B, 5E–G, 5O, 6A–C, 6H, 6M, 7A–B, S1, S2, S3, and S8.
